# Sustained Activation of CaMKII Promotes Skeletal Muscle Contractile Dysfunction in Aging

**DOI:** 10.1111/acel.70704

**Published:** 2026-09-10

**Authors:** Michael R. Bene, Tae Chung, Elizabeth D. Luczak, Gabriel Lopez‐Cecetaite, William A. Fountain, Giovanni Rosales‐Soto, Erick Hernández‐Ochoa, Corina Antonescu, Liliana Florea, Seeun J. Jeong, Emily Elassal, Anne Le, Qian‐Li Xue, Ahmet Hoke, Peter M. Abadir, Qinchuan Wang

**Affiliations:** ^1^ Division of Geriatric Medicine and Gerontology, Biology of Healthy Aging Program Johns Hopkins School of Medicine Baltimore Maryland USA; ^2^ Shirley Ryan AbilityLab Chicago Illinois USA; ^3^ Department of Physical Medicine and Rehabilitation Northwestern University Chicago Illinois USA; ^4^ Division of Cardiology Johns Hopkins School of Medicine Baltimore Maryland USA; ^5^ School of Health and Human Sciences Indiana University Indianapolis Indianapolis Indiana USA; ^6^ Department of Biochemistry and Molecular Biology, School of Medicine University of Maryland Baltimore Maryland USA; ^7^ Genetic Resources Core Facility, Computational Bioanalysis Division, and Department of Genetic Medicine Johns Hopkins School of Medicine Baltimore Maryland USA; ^8^ College of Computer, Mathematical, and Natural Sciences University of Maryland College Park Maryland USA; ^9^ Gigantest, Inc. Baltimore Maryland USA; ^10^ Department of Neurology and Neuroscience Johns Hopkins School of Medicine Baltimore Maryland USA

**Keywords:** calcium signaling, calcium‐calmodulin‐dependent protein kinase type 2, muscle weakness, oxidative stress, sarcopenia, skeletal muscle

## Abstract

Sarcopenia, the age‐related loss of muscle strength and mass, contributes to adverse health outcomes in older adults. Exercise engages calcium (Ca^2+^)‐ and redox‐dependent signaling pathways that enhance muscle performance and adaptation, whereas aging disrupts Ca^2+^ and redox homeostasis. CaMKII is a key transducer of both signals, raising the possibility that sustained CaMKII signaling becomes maladaptive with aging. Here, we show that CaMKII protein abundance is increased in aged mouse skeletal muscle and that sustained CaMKII activation in young muscle is sufficient to impair contractile function before substantial atrophy develops and, with prolonged activation, to promote progressive muscle loss. Sustained CaMKII activation also disrupted mitochondrial organization and shifted the young‐muscle transcriptome toward an aged profile characterized by inflammatory and stress‐response pathways. Inhibiting canonical NF‐κB signaling partially preserved contractile force during prolonged CaMKII activation without preserving muscle mass, and mediation analysis implicated heme/iron‐related transcriptional remodeling in the muscle‐mass‐independent decline in force. Conversely, expression of CN19o, a peptide inhibitor of CaMKII, in aged muscle improved contractile function and shifted the transcriptome away from an aging‐associated profile without inducing hypertrophy. Together, these findings identify sustained CaMKII signaling as a contributor to age‐associated muscle dysfunction and support a context‐dependent shift from adaptive CaMKII signaling in youth to maladaptive signaling in aging, consistent with antagonistic pleiotropy.

## Graphical abstract

CaMKII exemplifies antagonistic pleiotropy in skeletal muscle. Exercise‐related Ca^2+^/redox signals drive transient CaMKII activation supporting youthful performance. Here we show CaMKII is upregulated in aged muscle, where dysregulated Ca^2+^/redox signals likely sustain its activity; constitutively active CaMKII recapitulates aging‐associated contractile deficits, while CaMKII inhibition improves aged‐muscle function.

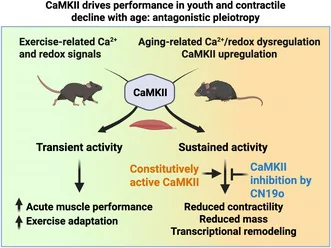

## 1 Introduction

Sarcopenia is a common age‐related disorder characterized primarily by low muscle strength and secondarily by low muscle mass or quality (Cruz‐Jentoft et al. 2019). It increases the risk of falls, disability, loss of independence, chronic diseases, and premature mortality, highlighting the need to identify molecular drivers of muscle aging and tractable therapeutic targets.

Age‐related decline in muscle function reflects multiple biological processes. Exercise training remains the first‐line intervention for the prevention and treatment of sarcopenia because it engages many of the pathways altered with aging (Sayer and Cruz‐Jentoft 2022; Granic et al. 2023). Exercise promotes muscle adaptation through coordinated molecular responses to repeated bouts of contractile activity (Egan and Sharples 2023). With repeated exercise, these responses enhance muscle performance and resilience.

Cytosolic calcium ions (Ca^2+^) regulate skeletal muscle contraction and also serve as signals for exercise‐induced metabolic and transcriptional adaptations. CaMKII is a key mediator of these Ca^2+^‐dependent signals in skeletal muscle (Chin 2005; Tavi and Westerblad 2011), and in young muscle contributes to acute contractile performance and exercise‐responsive metabolic and transcriptional adaptation (Wang et al. 2021; Tsukahara et al. 2026). Redox signaling constitutes a second major regulatory axis in exercising muscle. Reactive oxygen species and other redox‐active species can promote muscle adaptation through specific protein modifications (Powers et al. 2024), and we previously showed that redox‐dependent CaMKII activation in contracting skeletal muscle enhances both acute contractile performance and the transcriptional response to exercise (Wang et al. 2021).

Therefore, CaMKII mediates the physiological effects of Ca^2+^ and redox signals in exercising skeletal muscle to promote performance and adaptation. In young, healthy muscle, CaMKII activation scales with the intensity of contractile activity and resolves after exercise (Rose and Hargreaves 2003; Egan et al. 2010). In contrast, aged skeletal muscle exhibits redox dysregulation and altered intracellular Ca^2+^ handling, including oxidative and nitrosative modifications of RyR1, increased SR Ca^2+^ leak, reduced tetanic Ca^2+^ release, and impaired activity‐dependent mitochondrial Ca^2+^ signaling (Mecocci et al. 1999; Andersson et al. 2011; Pietrangelo et al. 2015; Lamboley et al. 2016). This dysregulated signaling environment may promote maladaptive engagement of CaMKII‐dependent pathways outside the normal exercise context. We therefore hypothesized that sustained, exercise‐independent CaMKII signaling contributes to skeletal muscle aging.

We first examined CaMKII abundance and autophosphorylation in skeletal muscle from aged mice. We then used AAV9‐mediated expression of constitutively active CaMKII as a gain‐of‐function approach to test whether sustained CaMKII signaling is sufficient to reduce muscle mass and impair muscle contractility. Conversely, an AAV9‐delivered CaMKII inhibitor, CN19o, was used to test whether CaMKII inhibition improves muscle function in aged mice. Together, these experiments support a model in which excessive CaMKII signaling contributes to skeletal muscle aging, including impaired contractile function, and with sustained activation, muscle atrophy. The CN19o experiment further suggests that CaMKII inhibition can improve contractile performance in aged muscle without increasing muscle mass, consistent with CaMKII acting as one component of the broader signaling network that shapes age‐related muscle dysfunction.

## 2 Results

### 2.1 CaMKII Abundance Is Increased in Skeletal Muscle of Older Mice

**FIGURE 1:**
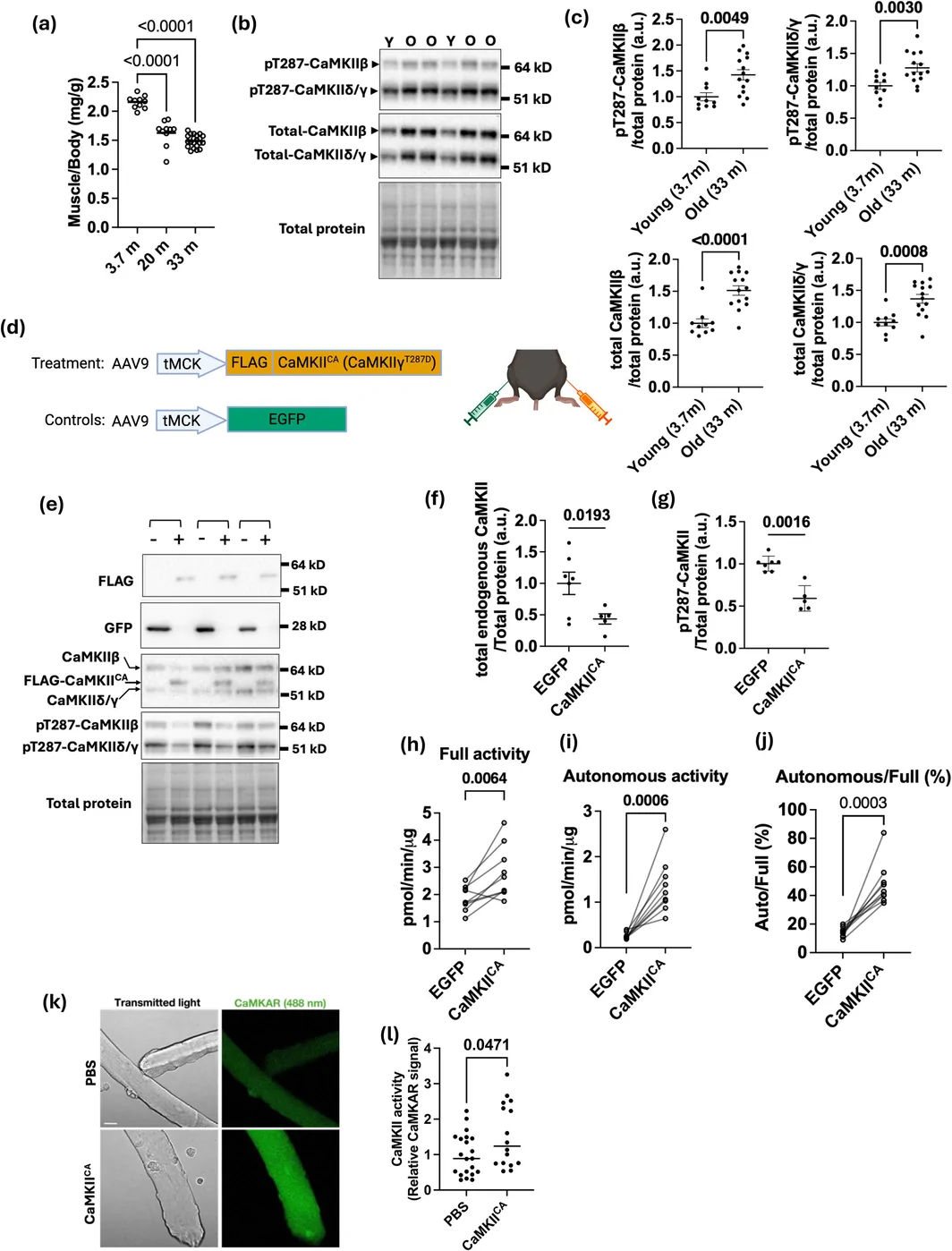
Aging is associated with reduced normalized TA mass and increased CaMKII abundance, and CaMKII^CA^ overexpression increases CaMKII activity in young skeletal muscle. (a) Tibialis anterior (TA) muscle mass normalized to body weight in young (3.7‐month‐old, *n* = 10; 5 male/5 female), old (20‐month‐old, *n* = 10; 5 male/5 female), and very old (33‐month‐old, *n* = 21; 21 male) mice. Each point represents one mouse. Data were analyzed by Welch's ANOVA followed by Dunnett's T3 multiple‐comparisons test. Exact *p* values are shown in the graph. (b) Representative Western blots of pT287‐CaMKII, total CaMKII, and total protein stain in young (Y, 3.7‐month‐old) and very old (O, 33‐month‐old) TA muscles. (c) Quantification of pT287‐CaMKIIβ, pT287‐CaMKIIδ/γ, total CaMKIIβ, and total CaMKIIδ/γ normalized to total protein stain in TA muscle. *n* = 10 young (3.7‐month‐old; 5 male/5 female) and *n* = 14 very old (33‐month‐old; 14 male) mice. Data were analyzed by Student's *t*‐test; *p* values are shown in the graphs. (d) Schematic of AAV9 constructs used for skeletal muscle gene delivery. FLAG‐tagged CaMKII^CA^ (CaMKIIγ‐T287D) or untagged enhanced green fluorescent protein (EGFP) was expressed under the muscle‐specific tMCK promoter. AAV9 vectors were injected into TA muscles, with mice receiving CaMKII^CA^ in one leg and EGFP in the contralateral leg for within‐animal comparisons. (e) Western blot analysis of protein extracted from injected TA muscles. Each bracket indicates one mouse; (+) denotes AAV9‐tMCK‐CaMKII^CA^ injection, and (−) denotes AAV9‐tMCK‐EGFP injection. Blots were probed with anti‐FLAG, anti‐EGFP, anti‐total CaMKII, and anti‐pT287‐CaMKII antibodies. (f, g) Quantification of endogenous total CaMKII and pT287‐CaMKII levels, combining CaMKIIβ and CaMKIIδ/γ signals. *n* = 7 EGFP‐expressing muscles and *n* = 5 CaMKII^CA^‐expressing muscles. Samples were from six mice: Five mice (3 male/2 female) expressed EGFP and CaMKII^CA^ in contralateral TA muscles, and one female mouse expressed EGFP in both TA muscles. Data were analyzed by Student's *t*‐test; *p* values are shown in the graphs. (h‐j) Radioactive CaMKII kinase assay measuring full CaMKII activity, autonomous CaMKII activity, and autonomous activity as a percentage of full activity in EGFP‐ and CaMKII^CA^‐expressing TA muscles. Paired TA muscles were collected from 9 mice (3 male/6 female) injected with AAV9‐tMCK‐EGFP in one TA and AAV9‐tMCK‐CaMKII^CA^ in the contralateral TA; EGFP *n* = 9 and CaMKII^CA^ *n* = 9. Data were analyzed by paired *t*‐test. (k) Confocal imaging of isolated single muscle fibers from flexor digitorum brevis (FDB) muscles expressing CaMKAR, a CaMKII activity reporter. Representative transmitted‐light and fluorescence (488 nm) images from PBS‐ and AAV9‐tMCK‐CaMKII^CA^‐injected muscles are shown. (l) Quantification of CaMKII activity based on CaMKAR signal intensity in isolated fibers. *n* = 21 PBS fibers and *n* = 16 CaMKII^CA^ fibers, isolated from three muscles per condition (3 males). Data were analyzed by Student's *t*‐test; the *p* value is shown in the graph.

We first assessed whether older mice used for age‐associated CaMKII protein and autophosphorylation measurements showed reduced skeletal muscle mass relative to body weight. Tibialis anterior (TA) muscle mass/body weight was significantly reduced in both 20‐month‐old and 33‐month‐old mice compared with 3.7‐month‐old mice (Figure 1a). Similar reductions were observed in gastrocnemius and quadriceps muscles, with the largest reductions in 33‐month‐old mice (Figure S1a). These data show that the aged mice used for the age‐related CaMKII analysis had reduced normalized skeletal muscle mass.

We next used Western blotting to assess CaMKII protein abundance and T287 autophosphorylation, the latter a biochemical marker of CaMKII activation (Brown and Bayer 2024), in TA muscles from young and very old mice. Total CaMKIIβ and CaMKIIδ/γ protein abundance were significantly increased in 33‐month‐old TA muscles compared with 3.7‐month‐old TA muscles (Figure 1b,c). pT287‐CaMKIIβ and pT287‐CaMKIIδ/γ signals were also significantly increased in these samples (Figure 1b,c). These data indicate that 33‐month‐old TA muscle has increased CaMKII protein abundance and elevated T287 autophosphorylation under resting conditions.

Additional measurements supported a robust increase in total CaMKII abundance across muscle groups in older mice, while suggesting that CaMKII autophosphorylation is more context‐dependent. In 20‐month‐old TA muscle and 33‐month‐old gastrocnemius muscle, total CaMKII abundance was increased relative to young muscle, whereas pT287‐CaMKII changes varied by isoform, age, and muscle type (Figure S1b,c). We therefore interpret increased CaMKII abundance as a reproducible feature of aged skeletal muscle, while recognizing that pT287‐CaMKII detected in whole‐muscle lysates may not be sufficient as a stand‐alone marker of chronic CaMKII activity, given that CaMKII is allosterically regulated by Ca^2+^/calmodulin and post‐translationally regulated by oxidation, nitrosylation, and O‐GlcNAcylation (Brown and Bayer 2024).

### 2.2 Adeno‐Associated Virus Serotype 9‐Mediated Expression of Constitutively Active CaMKII Increases CaMKII Activity in Skeletal Muscle

Because total CaMKII abundance was increased in aged muscle, but steady‐state biochemical markers such as pT287‐CaMKII may not fully capture chronic CaMKII signaling, we next used a gain‐of‐function approach to test the consequences of sustained CaMKII activation in skeletal muscle. Specifically, we used AAV9 to express FLAG‐tagged CaMKIIγ carrying a phosphomimetic T287D mutation that renders the kinase constitutively active (CaMKII^CA^) (Figure 1d). CaMKII^CA^ was driven by the muscle‐specific tMCK promoter to restrict transgene expression to muscle fibers (Wang et al. 2008). The CaMKII^CA^ construct was derived from CaMKIIγ transcript variant 2 (NM_001039138), the most abundant CaMKII isoform in skeletal muscle, as determined by our previously published transcriptomic data (Wang et al. 2021). AAV9‐tMCK‐CaMKII^CA^ was delivered via intramuscular injection into one TA muscle of each mouse, while the contralateral TA was injected with an equal viral load of AAV9‐tMCK‐Enhanced Green Fluorescent Protein (EGFP) as a control. This contralateral design enables paired comparisons within individual animals, thereby minimizing the influence of inter‐individual variability.

Western blotting confirmed the expression of the FLAG‐tagged CaMKII^CA^ and EGFP within the TA, with very little ectopic expression in the respective contralateral muscles, indicating that transgene expression remained localized to the injected muscles (Figure 1e). As anticipated, CaMKII^CA^ was detected using an anti‐total CaMKII antibody, and its expression level was comparable to that of endogenous CaMKII (Figure 1e). The pT287‐CaMKII antibody did not detect CaMKII^CA^, confirming its specificity, as the T287D mutation eliminates T287 phosphorylation. Notably, the expression and T287 phosphorylation of endogenous CaMKII were significantly reduced in CaMKII^CA^‐expressing muscles (Figure 1e–g), suggesting potential negative feedback mechanisms that limit CaMKII protein levels and autophosphorylation.

This reduction in endogenous total CaMKII and pT287‐CaMKII raised the question of whether net CaMKII enzymatic activity was nevertheless elevated in CaMKII^CA^‐expressing muscles. We therefore measured CaMKII activity using a CaMKII kinase activity assay with lysates from EGFP‐ and CaMKII^CA^‐expressing muscles. CaMKII^CA^ expression significantly increased full Ca^2+^/calmodulin‐dependent activity, Ca^2+^/calmodulin‐independent autonomous activity, and autonomous activity as a fraction of full activity (Figure 1h–j).

To confirm that CaMKII^CA^ also increases CaMKII activity within isolated muscle fibers, we measured CaMKII reporter activity in isolated flexor digitorum brevis (FDB) fibers. For this experiment, one FDB muscle was injected with AAV9‐tMCK‐CaMKII^CA^, while the contralateral FDB was injected with an equal volume of phosphate‐buffered saline (PBS) as the control. Both muscles were subsequently electroporated with a plasmid encoding the CaMKII activity reporter CaMKAR (Reyes Gaido et al. 2023), allowing CaMKII activity to be assessed by confocal imaging in isolated fibers (Figure 1k). PBS, rather than AAV9‐tMCK‐EGFP, was used as the control in this fluorescence‐based assay to avoid spectral overlap with the GFP‐derived CaMKAR reporter. CaMKAR signal was significantly higher in CaMKII^CA^‐expressing fibers than in control fibers (Figure 1l).

Together, the in vitro kinase activity assay and in cellulo CaMKAR reporter measurements show that AAV9‐tMCK‐CaMKII^CA^ increases net CaMKII activity in skeletal muscle despite reducing endogenous total CaMKII and pT287‐CaMKII. These results validate AAV9‐tMCK‐CaMKII^CA^ as a gain‐of‐function approach for testing the consequences of sustained CaMKII activity in skeletal muscle.

### 2.3 Sustained CaMKII Activation Progressively Weakens Tibialis Anterior Muscles and Induces Histological Remodeling

**FIGURE 2:**
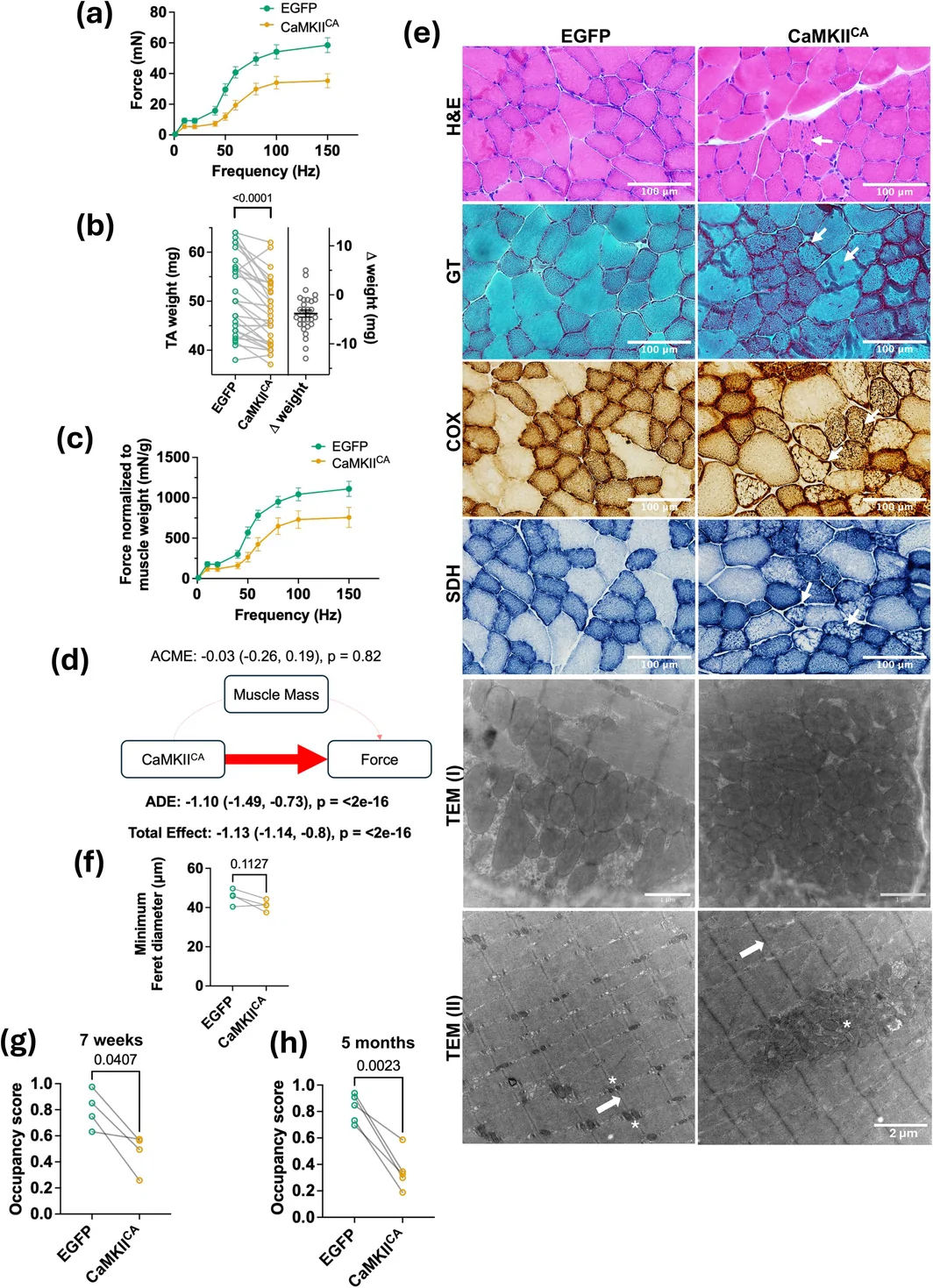
Sustained CaMKII activation impairs skeletal muscle contractility, reduces muscle mass, and disrupts muscle morphology. (a) In vivo isometric force‐frequency curves of TA muscles 6 weeks after contralateral injection in 9 mice (4 male/5 female) with AAV9‐tMCK‐EGFP in one TA and AAV9‐tMCK‐CaMKII^CA^ in the contralateral TA. *n* = 9 per condition. Data were analyzed by linear mixed‐effects model; estimate = −1.13, 95% CI [−1.44, −0.82], *p* = 1.44 × 10^−11^. (b) TA muscle weight 7 weeks post‐injection, shown as paired EGFP‐ and CaMKII^CA^‐expressing muscles with within‐animal weight differences. *n* = 30 paired TAs from 30 AAV9‐injected mice (15 male/15 female). Data were analyzed by paired Student's *t*‐test; the *p* value is shown in the graph. (c) Isometric force normalized to TA muscle weight. Same samples as in (a). Data were analyzed by linear mixed‐effects model; estimate = −1.87, 95% CI [−2.47, −1.28], *p* = 1.07 × 10^−4^. (d) Mediation analysis using the R mediation package to test whether reduced muscle mass mediated the effect of CaMKII^CA^ on force production. ACME, average causal mediation effect; ADE, average direct effect. (e) Representative H&E‐, modified Gomori's trichrome (GT)‐, COX‐, and SDH‐stained TA cross‐sections, and longitudinal TEM images from EGFP‐ and CaMKII^CA^‐expressing muscles. Histological sections and TEM (I) images were collected 7 weeks post‐injection; TEM (II) images are representative of the 5‐month post‐injection cohort. In histological images, arrows indicate abnormal staining patterns; in TEM images, arrows indicate Z‐lines, and asterisks indicate mitochondria. (f) Muscle fiber minimum Feret diameter from 4 paired mice (2 male/2 female). Each point represents the median of approximately 200 fibers from one muscle. *n* = 4 muscles per treatment. Data were analyzed by paired Student's *t*‐test; the *p* value is shown in the graph. (g, h) Z‐line mitochondrial occupancy score measured from longitudinal TEM images at 7 weeks (g; 4 paired mice, 2 male/2 female) and 5 months (h; 5 paired mice, 2 male/3 female) post‐injection. Z‐line mitochondrial occupancy was scored as 1, 0.5, or 0 when mitochondria were present at both, one, or neither end of each clearly defined Z‐line, respectively. Each point represents the mean occupancy score for all Z‐lines scored across multiple longitudinal TEM micrographs from one muscle. TEM micrographs were acquired by an operator blinded to treatment. Data were analyzed by paired Student's *t*‐test; *p* values are shown in the graphs.

Having established that CaMKII abundance is increased in aged skeletal muscle and that AAV9‐tMCK‐CaMKII^CA^ elevates net CaMKII activity in young muscle, we next assessed the functional impact of sustained CaMKII activation in vivo. To isolate these effects from age‐related confounders, we injected 3.5‐month‐old mice with AAV9‐tMCK‐CaMKII^CA^ in one TA and AAV9‐tMCK‐EGFP in the contralateral TA. Six weeks later, in vivo contractility was assessed by stimulating the peroneal nerve across a frequency range of 1–150 Hz in anesthetized mice. CaMKII^CA^‐expressing muscles produced significantly less force than their EGFP‐expressing counterparts across all frequencies (estimate = −1.13 [−1.44, −0.82], *p* = 1.44 × 10^−11^, Figure 2a). At the seven‐week endpoint, CaMKII^CA^‐expressing TAs weighed about 7.0% less than paired contralateral controls (*p* < 0.0001, Figure 2b), indicating a modest but significant reduction in muscle mass. When the measured forces were normalized against the muscle mass, the normalized force remained significantly smaller in CaMKII^CA^‐expressing muscles (estimate = −1.87 [−2.47, −1.28], *p* = 1.07 × 10^−4^, Figure 2c). Mediation analysis showed a significant direct effect of CaMKII^CA^ on contractility (Average Direct Effect [ADE]: −1.10, *p* ≤ 2 × 10^−16^, Figure 2d), while muscle mass did not significantly mediate this effect (Average Causal Mediation Effect [ACME]: −0.03, *p* = 0.82). Consequently, the Total Effect of CaMKII^CA^ on force (−1.13, *p* ≤ 2 × 10^−16^) was almost entirely attributed to its direct negative influence. Thus, sustained CaMKII activation for 6–7 weeks compromises muscle contractile performance by reducing intrinsic force‐generating capacity while causing a modest but significant reduction in muscle mass.

Because mediation analysis indicated that reduced muscle mass did not account for the CaMKII^CA^‐induced reduction in contractile force, we conducted histological and ultrastructural analyses to examine additional structural changes associated with impaired function (Figure 2e–h). At 7 weeks post‐injection, routine H&E staining revealed an absence of endomysial inflammation or necrotic fibers in CaMKII^CA^‐expressing TA muscles. Morphometric analysis of H&E‐stained muscle sections from four mice, each expressing CaMKII^CA^ in one TA and EGFP in the contralateral TA, revealed a non‐significant trend toward smaller minimum Feret diameters of muscle fibers in CaMKII^CA^‐expressing muscles (~200 fibers per muscle from the superficial and deep regions per section, paired *t*‐test, *p* = 0.1127; Figure 2f).

Some CaMKII^CA^‐expressing muscle fibers exhibited an altered basophilic staining pattern in H&E‐stained sections (Figure 2e, H&E panel, arrow). Modified Gomori's trichrome (GT) staining showed intensified subsarcolemmal and focal intrafiber staining in affected fibers (Figure 2e, GT panel, arrows). Histochemical staining for cytochrome c oxidase (COX) and succinate dehydrogenase (SDH) showed aggregated mitochondrial enzymatic activity in subsarcolemmal and internal regions of affected fibers (Figure 2e, COX and SDH panels, arrows). Consistent with the COX and SDH staining patterns, TEM images showed prominent subsarcolemmal mitochondrial aggregates in CaMKII^CA^‐expressing muscle at 7 weeks post‐injection (Figure 2e, TEM panel I) and prominent intermyofibrillar mitochondrial clumping at 5 months post‐injection (Figure 2e, TEM panel II). In longitudinal TEM sections, these mitochondrial aggregates were associated with disrupted regular alignment of intermyofibrillar mitochondria at the ends of Z‐lines (Figure 2e, TEM panel II). To quantify this altered mitochondrial organization, we defined a Z‐line mitochondrial occupancy score. Each clearly defined Z‐line was scored as 1 if mitochondria were present at both ends of the Z‐line, 0.5 if mitochondria were present at one end, and 0 if mitochondria were absent from both ends. CaMKII^CA^‐expressing muscles showed significantly lower Z‐line mitochondrial occupancy scores than EGFP‐expressing controls at both 7 weeks and 5 months after viral injection (Figure 2g,h).

To assess the long‐term consequences of sustained CaMKII activation, a separate cohort of mice injected with AAV9‐tMCK‐CaMKII^CA^ in one TA and PBS in the contralateral TA at 3.5 months of age was evaluated 9 months post‐injection. This cohort was established before the EGFP control virus became available. Nine months after injection, CaMKII^CA^‐expressing muscles exhibited impaired contractile force compared with the PBS‐injected contralateral controls (estimate = −0.99 [−1.39, −0.59], *p* = 4.21 × 10^−6^, Figure S2a). Reduction in muscle mass progressed from 7.0% at 7 weeks after CaMKII^CA^ expression to 24.2% 9 months after injection (Figure S2b). Thus, sustained CaMKII activation produces both functional impairment and progressive loss of muscle mass, with long‐term CaMKII^CA^ expression producing an atrophic phenotype comparable in magnitude to the age‐associated reduction in muscle mass measured in our cohorts (Figures 1a and S1a). Contractile force of CaMKII^CA^‐expressing muscles remained significantly smaller after normalization to muscle mass (estimate = −0.99, 95% CI [−1.39, −0.59], *p* = 4.21 × 10^−6^, Figure S2c). However, in contrast to the 6‐week functional assessment, when loss of force was not significantly mediated by reduced muscle mass, mediation analysis at 9 months showed that the decline in contractile force was explained by loss of muscle mass (ACME: −1.24, *p* ≤ 2 × 10^−16^, Figure S2d).

Building on the structural alterations observed at 7 weeks and considering the more pronounced functional decline and atrophy at 9 months, we examined muscle histology to evaluate potential progression of tissue remodeling (Figure S2e–g). H&E staining revealed numerous atrophic fibers in CaMKII^CA^‐expressing muscles and the absence of endomysial inflammation or necrotic fibers (Figure S2e, H&E panel). Morphometric analysis revealed a significant reduction in the minimum Feret diameter of fibers in CaMKII^CA^ muscles compared to contralateral controls (*p* = 0.0143; Figure S2f). A pooled visualization showed that CaMKII^CA^‐expressing muscles had a leftward shift in the diameter distribution (Figure S2g).

Compared with the 7‐week findings, GT staining at 9 months showed more pronounced changes in staining patterns (Figure S2e, GT panel). COX staining revealed extensive changes in mitochondrial distribution, with preserved COX activity (Figure S2e, COX panel). Despite the extensive remodeling of mitochondrial organization, CaMKII^CA^ did not measurably alter mitochondrial protein content relative to total muscle protein. Immunoblotting for representative subunits of respiratory‐chain complexes I–V revealed no significant difference in their steady‐state abundance at either 7 weeks (Figure S3a,b) or 9 months (Figure S4a,b) after viral injection. This included nuclear‐encoded subunits (NDUFB8, SDHB, UQCRC2, ATP5A) and the mitochondrial‐encoded subunit MTCO1, indicating preserved stoichiometric balance between nuclear‐ and mitochondrial genome‐encoded components of the respiratory chain complexes. Consistently, quantitative PCR showed an unchanged mitochondrial‐to‐nuclear DNA copy number ratio at 7 weeks (Figure S3c). These findings suggest that CaMKII^CA^ alters mitochondrial spatial organization without affecting the abundance of mitochondrial constituents in skeletal muscle.

### 2.4 Chronic CaMKII Activation Shifts the Transcriptome of Young Muscle Toward an Aged Profile, Whereas CN19o Expression Modestly Opposes Age‐Associated Transcriptomic Changes

**FIGURE 3:**
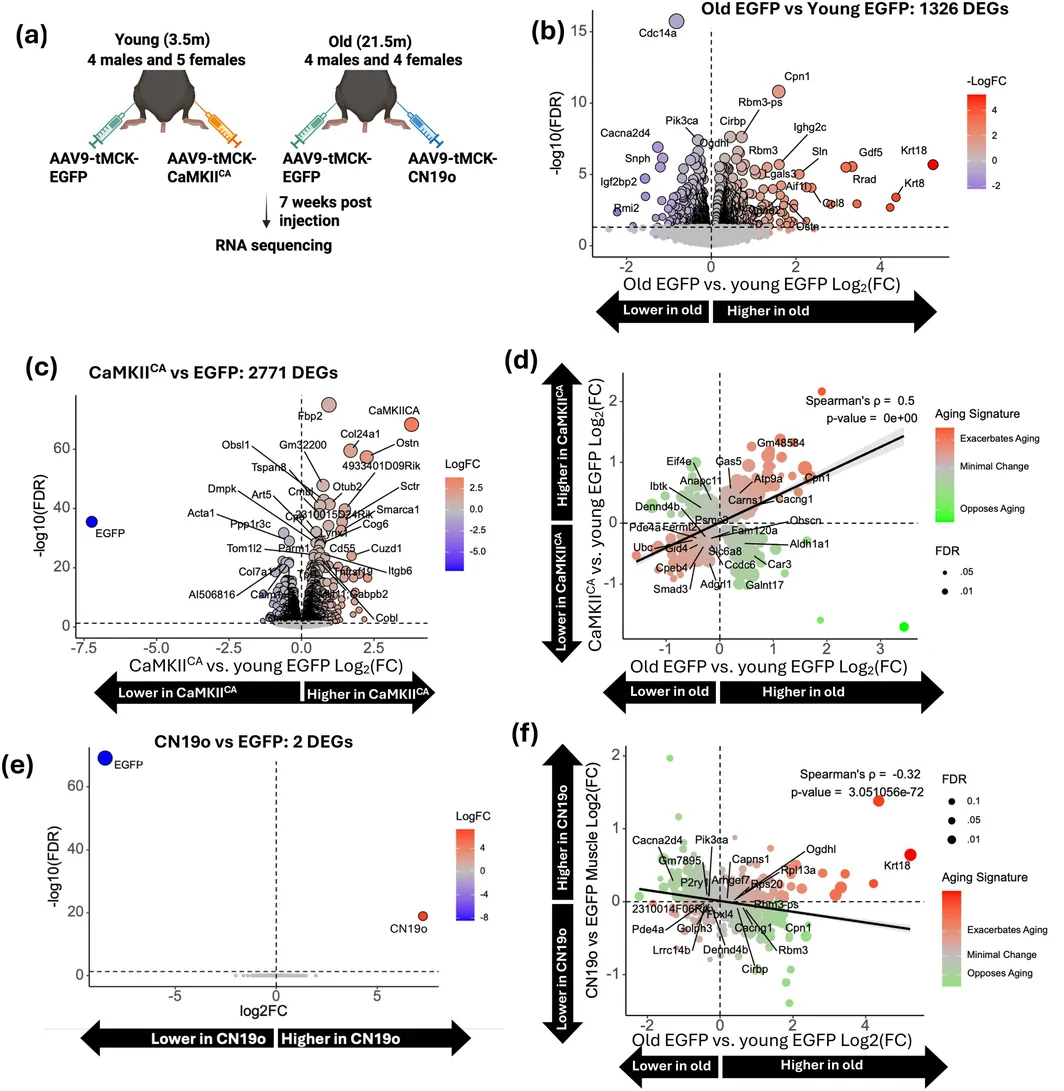
Constitutively active CaMKII (CaMKII^CA^) shifts the young muscle transcriptome toward an older profile, while CaMKII inhibition by CN19o partially rejuvenates the transcriptome of aged muscles. (a) Experimental design. Young mice (3.5 months, *n* = 9, 4 males and 5 females) were injected with AAV9‐tMCK‐EGFP in one TA and AAV9‐tMCK‐CaMKII^CA^ in the contralateral TA. Old mice (21.5 months, *n* = 8, 4 males and 4 females) were injected with AAV9‐tMCK‐EGFP in one TA and AAV9‐tMCK‐CN19o (CaMKII inhibitory peptide) in the contralateral TA. TA muscles were collected 7 weeks post‐injection and subjected to RNA sequencing. (b) Volcano plot comparing gene expression between old versus young EGFP‐expressing TA muscles; differentially expressed genes (DEGs). (c) Volcano plot comparing gene expression between young CaMKII^CA^‐ and young EGFP‐expressing TA muscles. (d) Correlation analysis between genes regulated by aging (old vs. young EGFP) and by CaMKII^CA^ expression (young CaMKII^CA^ vs. young EGFP). Color indicates whether CaMKII^CA^ promoted, opposed, or minimally influenced aging‐like changes of gene expression. Spearman's ρ and *p* value are shown. A linear trendline was included as a visual guide to indicate the overall correlation. (e) Volcano plot comparing gene expression between old CN19o‐ and old EGFP‐expressing TA muscles. (f) Correlation analysis between genes regulated by aging (old vs. young EGFP) and by CN19o expression (old CN19o vs. old EGFP). Color indicates whether CN19o promoted, opposed, or minimally influenced aging‐like changes of gene expression. Spearman's ρ and *p* value are shown. A linear trendline was included as a visual guide to indicate the overall correlation.

Sustained CaMKII activation impairs muscle function, alters fiber morphology, and disrupts mitochondrial organization, suggesting that chronic CaMKII signaling may also promote transcriptional remodeling. To test this, we performed poly(A) RNA sequencing (RNA‐seq) on TA muscles 7 weeks after in vivo modulation of CaMKII activity (Figure 3a). In young adult mice (3.5 months; 4 males, 5 females), one TA was injected with AAV9‐tMCK‐CaMKII^CA^ and the contralateral TA with AAV9‐tMCK‐EGFP, enabling within‐subject comparisons between CaMKII^CA^‐expressing and control muscles, independent of aging‐related confounders. The seven‐week time point was chosen to capture transcriptomic changes associated with early functional and histological alterations, before the extensive structural degeneration observed at later stages. In parallel, aged mice (21.5 months; 4 males, 4 females) were injected with AAV9‐tMCK‐CN19o in one TA and AAV9‐tMCK‐EGFP in the contralateral TA to assess the effects of CN19o‐mediated CaMKII inhibition in aged muscle. CN19o is a well‐characterized peptide inhibitor of CaMKII (Coultrap and Bayer 2011).

Differential expression analysis identified 1326 differentially expressed genes (DEGs) between old versus young EGFP‐expressing TAs (Figure 3b). Seven weeks of CaMKII^CA^ expression in young muscle elicited an even broader response, with 2771 genes differentially expressed relative to contralateral EGFP controls (Figure 3c). To determine how closely CaMKII^CA^ expression resembles the aging transcriptional program, we correlated the Log_2_(fold changes) for all genes in the two comparisons (young CaMKII^CA^ vs. young EGFP and old EGFP vs. young EGFP). The correlation showed a moderate but highly significant concordance (Spearman's ρ = 0.50, *p* ≈0; Figure 3d). Of the 1326 genes significantly altered with age, 73.5% were regulated in the same direction by CaMKII^CA^, while 26.5% showed discordant regulation. Conversely, 69.9% of the 2771 CaMKII^CA^‐regulated genes were shifted in the same direction by aging, while 30.1% were discordant. These data indicate that chronic CaMKII activation shifts the young‐muscle transcriptome toward an aging‐associated profile while also driving distinct gene‐expression changes, suggesting that additional pathways cooperate with CaMKII or act independently to shape the full breadth of age‐related transcriptomic remodeling.

In aged TA muscles, AAV‐driven expression of the CaMKII inhibitor CN19o resulted in only a minimal number of statistically significant transcriptional changes: after false‐discovery correction (FDR *q* < 0.05), only the two transgene mRNAs, CN19o and EGFP, met the statistical threshold for differential expression (Figure 3e). This muted response may reflect the low transcript abundance of CN19o relative to endogenous CaMKII isoforms (mean transcript length‐normalized counts ± SEM: CN19o 0.56 ± 0.15; *Camk2b* 1.40 ± 0.13; *Camk2d* 0.22 ± 0.03; *Camk2g* 0.89 ± 0.05), but the extent of biochemical CaMKII inhibition cannot be inferred directly from RNA abundance.

Although few individual genes crossed the FDR threshold, CN19o nonetheless shifted the global expression pattern in the opposite direction from the transcriptional changes observed with aging. Spearman's correlation of Log_2_(fold changes) for all genes revealed a significant inverse relationship between CN19o‐ and aging‐associated expression changes (ρ = −0.32, *p* = 3.05 × 10^−72^; Figure 3f). This broad but modest effect suggests that CN19o expression partially offsets age‐associated transcriptomic drift through coordinated, low‐magnitude changes across many genes.

### 2.5 Sustained CaMKII Activation Recapitulates Aging‐Associated Pathway Signatures

**FIGURE 4:**
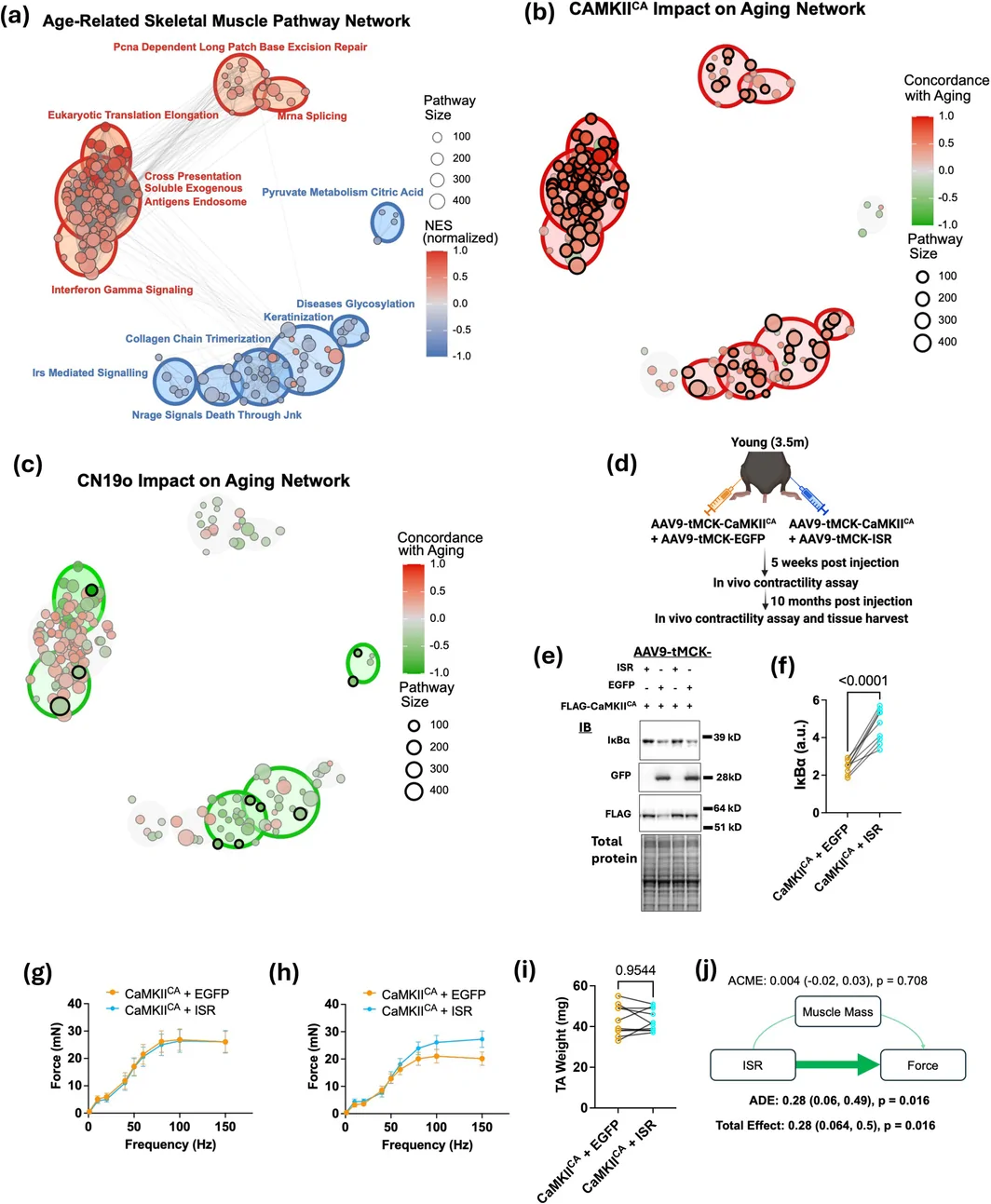
CaMKII activation drives aging‐related pathway changes in skeletal muscle; its inhibition by CN19o reverses some inflammatory signatures in aged muscle, and ISR‐mediated blockade of canonical NF‐κB signaling partially preserves contractile force during long‐term CaMKII^CA^ expression. (a) Network plot of Reactome pathways that are significantly changed in old EGFP versus young EGFP (FDR < 0.05), defined as Aging Network. Of the 190 total pathways identified, 135 were upregulated, and 55 were downregulated; clusters are labeled by the representative pathway with the greatest within‐cluster and the least between‐cluster gene sharing. (b) Aging Network plot with pathways colored by concordance/discordance between aging effect and CaMKII^CA^ effect; 178 CaMKII^CA^ pathways are concordant with the Aging Network, 12 discordant. Pathways with FDR < 0.05 in CaMKII^CA^ (124 pathways) are labeled with bold outlines. (c) Aging Network plot with pathways colored by concordance/discordance between aging effect and CN19o effect; 95 CN19o pathways are concordant with Aging Network, and 95 discordant. Pathways with FDR < 0.05 in CN19o (10 pathways) are labeled with bold outlines. (d) Schematic of the experimental design testing whether IκBα super‐repressor (ISR, S32A/S36A‐IκBα) modifies CaMKII^CA^‐induced muscle dysfunction. AAV9‐tMCK‐CaMKII^CA^ was injected into both TA muscles of young mice (4 males/5 females), with AAV9‐tMCK‐EGFP co‐injected into one TA and AAV9‐tMCK‐ISR co‐injected into the contralateral TA. (e) Western blot validation of ISR, EGFP, and FLAG‐CaMKII^CA^ expression in injected TA muscles, collected 10 months after viral injection. ISR was detected by anti‐IκBα and co‐migrates with endogenous IκBα; total protein staining is shown as a loading reference. (f) Quantification of the anti‐IκBα band shown in panel (e). Each line connects paired muscles from the same mouse. Data were analyzed by paired Student's *t*‐test; *p* value is shown. (g) In vivo isometric force‐frequency curves from TA muscles co‐expressing CaMKII^CA^ with EGFP or ISR, measured 5 weeks post‐injection of AAV9‐tMCK‐CaMKII^CA^ together with either AAV9‐tMCK‐EGFP or AAV9‐tMCK‐ISR. *n* = 9 per condition; the two conditions did not differ significantly at this early time point (*p* = 0.1). (h) In vivo isometric force‐frequency curves of the same group of mice measured 10 months post‐injection. ISR co‐expression resulted in significantly greater contractile force than in the paired CaMKII^CA^ + EGFP control, consistent with partial preservation of long‐term force. Data were analyzed by linear mixed‐effects model; estimate = 0.39, 95% CI [0.18, 0.59], *p* = 3.22 × 10^−4^. (i) TA muscle weight 10 months after co‐injection of CaMKII^CA^ with EGFP or ISR. Each line connects paired muscles from the same mouse. *p* value shown in the graph, *n* = 10 pairs. (j) Mediation analysis using the R mediation package. ACME, average causal mediation effect; ADE, average direct effect.

Because the single‐gene analysis indicated that CaMKII^CA^ shifts young muscle toward an aging‐associated expression profile, whereas CN19o modestly opposes age‐associated transcriptomic drift, we next asked which biological pathways underlie these coordinated changes. We therefore performed gene set enrichment analysis (Korotkevich et al. 2021) using Reactome canonical pathways (Milacic et al. 2024). The normalized enrichment score (NES) was used to indicate the direction and magnitude of pathway enrichment, and the old EGFP versus young EGFP comparison was used as the aging‐associated pathway backdrop for interpreting the CaMKII^CA^ and CN19o effects. In old EGFP‐injected muscle compared with young EGFP‐injected muscle, 190 Reactome pathways met FDR < 0.05, including 135 pathways positively enriched with aging and 55 pathways negatively enriched with aging (Figure 4a and Table S1). Aging was associated with positive enrichment of immune and stress‐response pathway clusters, including antigen processing and cross‐presentation, interferon‐gamma signaling, eukaryotic translation elongation, mRNA splicing, and PCNA‐dependent long‐patch base excision repair. In contrast, negatively enriched age‐associated clusters included insulin receptor signaling, collagen chain trimerization and extracellular matrix‐related pathways, NRAGE/JNK‐associated signaling, glycosylation/keratinization, and pyruvate/citric acid cycle metabolism. Thus, the Reactome analysis identified coordinated age‐associated activation of immune, RNA‐processing, DNA‐repair, and protein‐translation programs together with reduced enrichment of metabolic and structural‐remodeling pathways.

We next asked whether sustained CaMKII activation shifted these age‐associated pathways in the same direction as aging. To do this, we overlaid the young CaMKII^CA^ versus young EGFP enrichment results onto the 190 Reactome pathways significantly altered by aging and colored each pathway by its concordance with the aging effect. Among these age‐associated pathways, 178 showed CaMKII^CA^‐associated NES values in the same direction as aging, whereas 12 showed NES values in the opposite direction; 124 pathways were also significantly altered by CaMKII^CA^ (Figure 4b and Table S1). Concordant CaMKII^CA^‐associated changes spanned most age‐modulated pathway clusters, including immune/endosomal antigen‐presentation pathways, interferon‐gamma signaling, translation elongation, mRNA splicing, DNA repair, collagen and extracellular‐matrix‐related pathways, glycosylation/keratinization, and NRAGE/JNK‐associated signaling. These data indicate that sustained CaMKII pathway engagement in young muscle produces a broad shift toward the aging‐associated Reactome pathway landscape.

We then evaluated whether CN19o expression in aged muscle shifted these age‐associated pathways in the opposite direction from aging. In contrast to the broad CaMKII^CA^ effect, only 10 of the 190 age‐associated Reactome pathways were also significantly altered by CN19o (Figure 4c and Table S1). Across all 190 age‐associated pathways, CN19o‐associated NES values were evenly split: 95 were in the same direction as aging, whereas 95 were in the opposite direction. Among the pathways significantly altered by CN19o, those that shifted in the opposite direction of aging were found in clusters related to eukaryotic translation elongation, interferon‐gamma signaling, collagen chain trimerization, keratinization, and pyruvate/citric acid cycle metabolism. These findings suggest that CN19o produces a more limited transcriptomic effect than CaMKII^CA^, but one that modestly opposes selected components of the aging‐associated pathway signature.

Together, the gene set enrichment analysis indicates that aging is accompanied by coordinated remodeling of immune, proteostasis, RNA‐processing, DNA‐repair, metabolic, and extracellular‐matrix pathway programs. Sustained CaMKII activation matches the direction of many of these age‐associated pathway changes, whereas CN19o expression in aged muscle produces a smaller and more selective shift that is partly opposite to aging. These pathway‐level results extend the single‐gene analysis by showing that CaMKII^CA^ broadly aligns with aging‐associated transcriptomic remodeling, whereas CN19o modestly opposes selected components of it.

### 2.6 NF‐κB Blockade Partially Preserves Long‐Term Contractile Force Independently of Muscle Mass

Our pathway analysis showed that inflammatory pathways were activated by both aging and CaMKII^CA^ expression and modestly opposed by CN19o. Chronic inflammation contributes to sarcopenia (Granic et al. 2023), and NF‐κB activation promotes muscle wasting (Cai et al. 2004). Because CaMKII can activate NF‐κB signaling (Martin et al. 2018), we asked whether blocking canonical NF‐κB signaling could attenuate CaMKII^CA^‐induced contractile dysfunction and whether any functional benefit was mediated by changes in muscle mass.

To test this pathway directly, we injected AAV9‐tMCK‐CaMKII^CA^ into both TAs of 3.5‐month‐old mice, with one TA co‐injected with AAV9‐tMCK‐IκBα‐super‐repressor (ISR) and the contralateral TA co‐injected with AAV9‐tMCK‐EGFP as a paired control (Figure 4d). ISR is a degradation‐resistant IκBα mutant carrying S32/36A substitutions and has been shown to inhibit NF‐κB signaling and attenuate muscle wasting in denervation and cancer‐cachexia models (Cai et al. 2004). Ten months after injection, ISR‐injected TAs had significantly higher IκBα protein abundance than paired control TAs (Figure 4e,f), consistent with sustained ISR expression. At 5 weeks post‐injection, force generation did not differ significantly between CaMKII^CA^ + EGFP and CaMKII^CA^ + ISR muscles (Figure 4g), indicating no detectable early benefit of ISR. At 10 months, however, ISR co‐expression resulted in significantly greater force than in the paired CaMKII^CA^ + EGFP control, partially preserving long‐term contractile function (Figure 4h; linear mixed‐effects model; estimate = 0.39, 95% CI [0.18, 0.59], *p* = 3.22 × 10^−4^). This protection was not accompanied by preservation of muscle size, as TA mass did not differ significantly between CaMKII^CA^ + EGFP and CaMKII^CA^ + ISR muscles (Figure 4i), and mediation analysis indicated that the effect of ISR on force was not mediated by muscle mass (Figure 4j). Thus, ISR‐mediated blockade of canonical NF‐κB signaling did not detectably improve the early CaMKII^CA^‐induced force deficit but partially preserved long‐term force independently of muscle mass.

### 2.7 The Heme Metabolism Gene Set Enrichment Score Is a Principal Transcriptomic Correlate of CaMKII^CA^ ‐Induced Muscle Weakness

**FIGURE 5:**
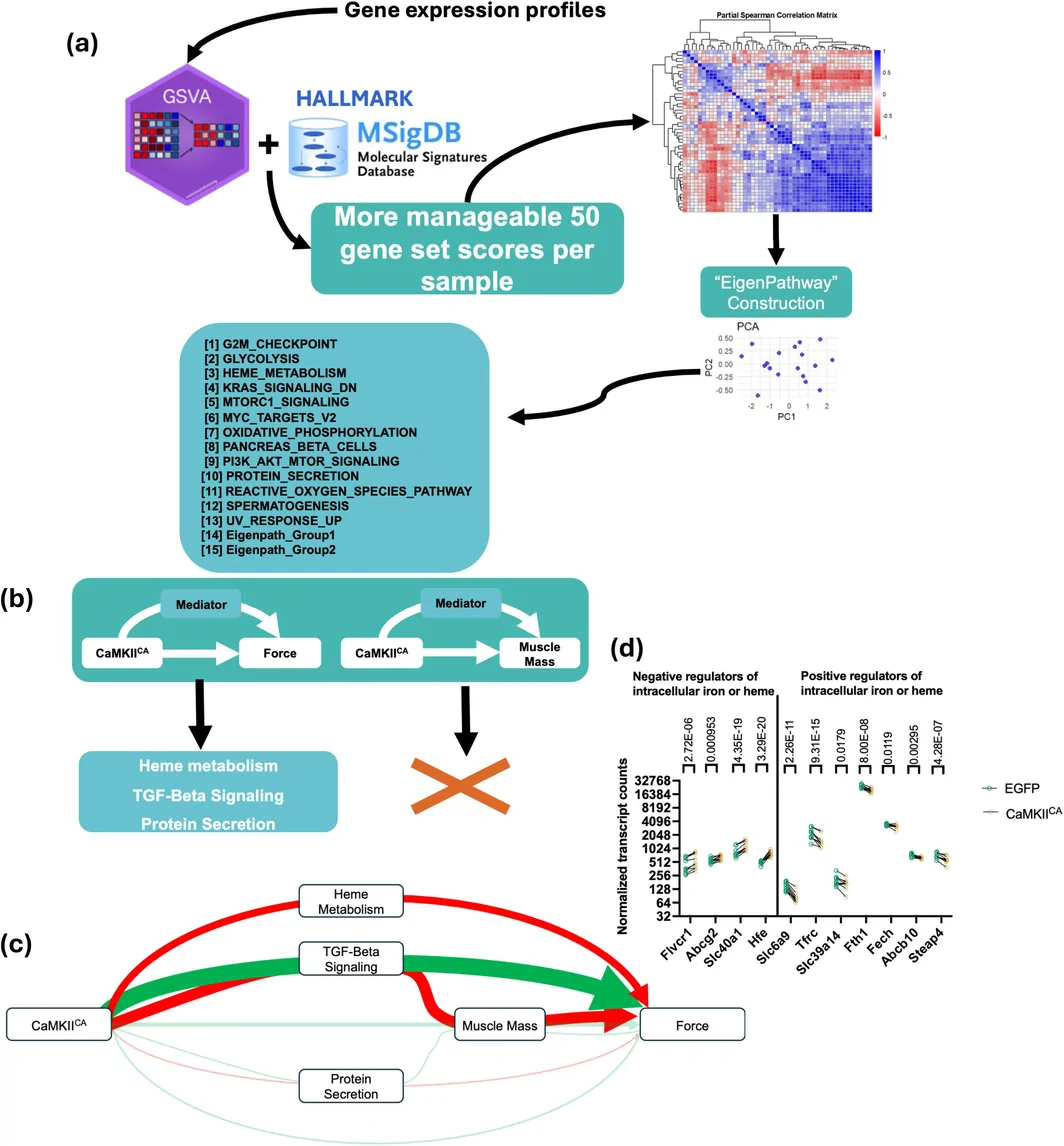
Gene set variation analysis (GSVA) and mediation analysis identify heme metabolism as a direct mediator of CaMKII^CA^‐induced muscle weakness. (a) To identify potential mediating effects in the transcriptome, GSVA was used to derive enrichment scores for the 50 Hallmark gene sets in each sample. Partial Spearman correlation analysis was performed on the gene set enrichment scores, adjusting for subject ID, to identify redundant gene sets, defined as those with |ρ| ≥ 0.8, with two clusters identified and reduced to “eigenpathways” based on principal component analysis with the first principal component representing the new “eigenpathway” score. (b) All potential mediators were evaluated through two mediation models, one mediating contractile force and another mediating muscle mass, using the “mediation” package in R. After correcting for multiple comparisons, three significant mediators of contractile force were identified, and no significant mediator of muscle mass. (c) Diagram of the full structural equation model (SEM). Each arrow represents a path from CaMKII^CA^ treatment (independent variable) to contractile force (outcome), with boxes representing mediators of paths. The color and size of the arrows denote effect (red negative, green positive, and size magnitude), and the opaque arrows indicate significant paths. (d) Normalized transcript counts of heme and iron regulators. Whole transcriptome multiple‐test adjusted *p* values from DESeq2 analysis are included in the figure.

The partial force protection conferred by ISR supports a contribution of canonical NF‐κB signaling to the long‐term contractile dysfunction caused by CaMKII^CA^. However, the incomplete force protection and the absence of muscle mass preservation indicate that additional downstream mechanisms are involved. To identify transcriptomic candidates that might contribute to these remaining effects, we leveraged matched transcriptomic, mass, and force measurements from individual muscles for mediation analysis. Given the high dimensionality of transcriptomic data, mediation analysis at the individual gene level was not feasible. To address this, we first applied gene set variation analysis (GSVA) (Hänzelmann et al. 2013), a single‐sample gene set enrichment method, to derive enrichment scores for the 50 Hallmark gene sets to reduce dimensionality (Figure 5a) (Liberzon et al. 2015). Despite the low redundancy among these curated gene sets, enrichment scores were strongly correlated across many of them (ρ ≥ 0.8; Figure 5a), indicating shared underlying transcriptional activity.

To minimize redundancy, we aggregated highly correlated gene sets (ρ ≥ 0.8) into composite variables termed “eigenpathways.” This yielded 15 candidate gene sets, 13 from the original Hallmark collection and two composite eigenpathways (see Table S2 for their compositions) for mediation analysis. Each was evaluated as a potential mediator of CaMKII^CA^'s effects on muscle mass and contractile force. No gene set significantly mediated the effect on muscle mass following correction for multiple comparisons. In contrast, three gene sets, heme metabolism, TGF‐β signaling, and protein secretion, significantly mediated the reduction in contractile force (Figure 5b).

To integrate these findings, we constructed a structural equation model (SEM) in which heme metabolism, TGF‐β signaling, and protein secretion were entered as parallel mediators of CaMKII^CA^ effects on muscle mass and contractile force. The initial model allowed each gene set to have only a direct path to force, independent of muscle mass. Indirect paths through muscle mass were subsequently included when the modification index exceeded 3.84, indicating a statistically significant improvement in model fit. In the final SEM, heme metabolism retained a single direct path to force, whereas TGF‐β signaling and protein secretion showed both direct and muscle mass‐mediated paths to force (Figure 5c). TGF‐β signaling exhibited an inconsistent mediation pattern, where a significant negative indirect effect on force via reduced muscle mass was partially offset by a smaller positive direct effect. Protein secretion did not retain significant mediation effects in the full model.

Collectively, these results identify the GSVA enrichment score of the heme metabolism gene set as the principal transcriptomic mediator of CaMKII^CA^‐induced contractile impairment, independent of muscle mass loss. Given this statistical association and the close relationship between heme metabolism and iron homeostasis (Dutt et al. 2022), we examined the expression of key genes related to heme and iron regulation in the RNA‐seq data. We found that CaMKII^CA^ significantly altered transcripts involved in both heme and iron handling. Genes associated with heme/iron export or homeostatic restriction were upregulated, including the heme/porphyrin exporters *Flvcr1* and *Abcg2*, the iron exporter *Slc40a1* (ferroportin), and *Hfe*. In contrast, genes supporting heme synthesis, mitochondrial heme handling, iron uptake, or iron storage were downregulated, including *Slc6a9*, which imports glycine for heme synthesis; *Fech*, the terminal enzyme in heme biosynthesis; *Abcb10*, a mitochondrial transporter linked to heme biogenesis; the iron importers *Tfrc* and *Slc39a14*; the iron‐handling metalloreductase *Steap4*; and the iron storage protein *Fth1* (Figure 5d).

To identify candidate transcriptional regulators associated with the gene‐expression changes induced by CaMKII^CA^, we used the CollecTRI regulatory network to infer transcription factor activity from the differential expression of their target genes (Müller‐Dott et al. 2023). Spic was the top‐ranked activated transcription factor in CaMKII^CA^‐expressing young muscle (Figure S5a). Spic is a heme‐responsive transcription factor that promotes the development of iron‐recycling macrophages, where heme induces Spic expression by promoting degradation of its transcriptional repressor Bach1 (Haldar et al. 2014). Conversely, Spic was the top‐ranked suppressed transcription factor in CN19o‐expressing aged muscle (Figure S5b). This reciprocal pattern across the CaMKII gain and reduction‐of‐function comparisons converges with the heme/iron‐associated expression changes described above and identifies Spic‐associated regulatory activity as a candidate link between CaMKII signaling and heme/iron‐related transcriptional remodeling.

### 2.8 Inhibiting CaMKII in Aged Muscles Improves Contractility Without Inducing Hypertrophy

**FIGURE 6:**
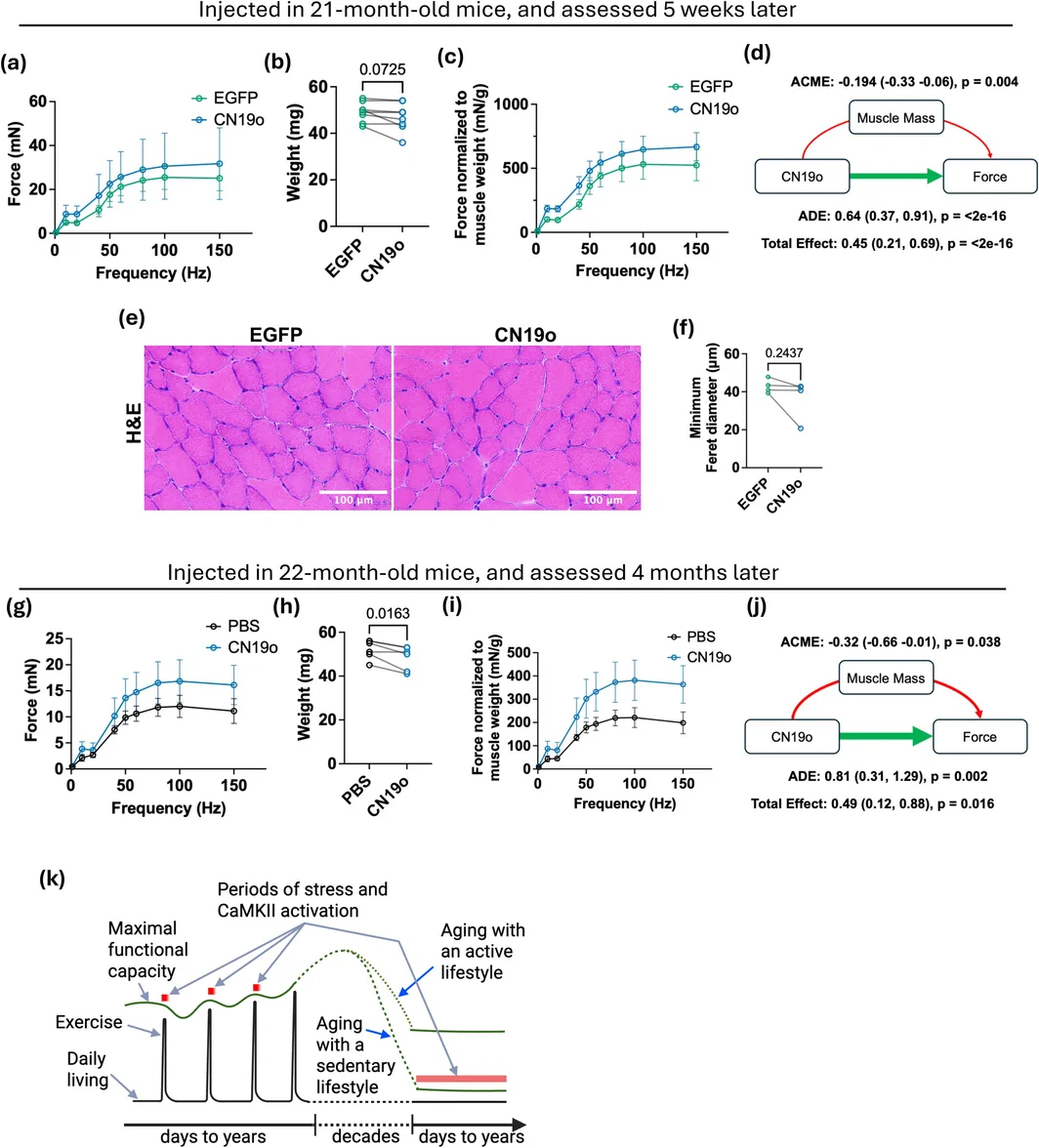
Inhibiting CaMKII in aged muscles improves contractility without inducing hypertrophy. (a) In vivo isometric force‐frequency curves of TA muscles 5 weeks after injection with AAV9‐tMCK‐CN19o in one TA and AAV9‐tMCK‐EGFP in the contralateral TA. *n* = 8 mice (4 male/4 female), with paired contralateral TA muscles; 8 muscles per condition. Data were analyzed by linear mixed‐effects model; estimate = 0.45 95% CI [0.2, 0.7], *p* = 5.04 × 10^−4^. (b) TA muscle mass 5 weeks post‐injection. Same muscles as in (a), *n* = 8 pairs; paired Student's *t*‐test, *p* value in graph. (c) Isometric force normalized to TA mass. Linear mixed‐effects model, estimate = 0.79 95% CI [0.42, 1.17], *p* = 5.09 × 10^−5^. (d) Mediation analysis using the R mediation package. ACME, average causal mediation effect; ADE, average direct effect. (e) Representative H&E staining of TA cross‐sections 6 weeks post‐injection. (f) Muscle fiber minimum Feret diameter from 4 old mice (2 male/2 female), with paired contralateral TA muscles. Each point represents the median of approximately 200 fibers from one muscle. *n* = 4 muscles per treatment. Data were analyzed by paired Student's *t*‐test; the *p* value is shown in the graph. (g) In vivo isometric force‐frequency curves of TA muscles 4 months after injection with AAV9‐tMCK‐CN19o in one TA and PBS in the contralateral TA. *n* = 6 old mice (4 male/2 female), with paired contralateral TA muscles; 6 muscles per condition. Data were analyzed by linear mixed‐effects model; estimate = 0.5, 95% CI [0.11, 0.89], *p* = 0.0131. (h) TA muscle mass 4 months post‐injection. *n* = 6 paired TA muscles from 6 old mice (4 male/2 female). Data were analyzed by paired Student's *t*‐test; the *p* value is shown in the graph. (i) Isometric force normalized to TA muscle mass. Same mice as in (g). Data were analyzed by linear mixed‐effects model; estimate = 0.96, 95% CI [0.33, 1.59], *p* = 0.0033. (j) Mediation analysis using the R mediation package. (k) Conceptual model. Green lines and green dotted lines indicate maximal functional capacity; the black curve indicates functional demand from daily living and exercise; red blocks indicate stress‐induced CaMKII activation. See Discussion for details.

To test whether the transcriptomic rejuvenation induced by CN19o translates into functional benefit, we injected AAV9‐tMCK‐CN19o in one TA of 21‐month‐old mice and AAV9‐tMCK‐EGFP in the contralateral TA. Five weeks later, CN19o‐expressing muscles generated higher absolute force across the 1–150 Hz stimulation range (linear mixed‐effect model, estimate = 0.45 [0.2, 0.7], *p* = 5.04 × 10^−4^, Figure 6a). This functional enhancement occurred despite a trend toward reduced muscle mass in the CN19o‐treated TAs (~5% reduction vs. EGFP, *p* = 0.0725; Figure 6b). Consequently, when force was normalized to muscle mass, CN19o‐expressing muscles demonstrated a pronounced improvement (linear mixed‐effect model, estimate = 0.79 [0.42, 1.17], *p* = 5.09 × 10^−5^, Figure 6c). Mediation analysis confirmed a significant direct positive effect of CN19o on force (Average Direct Effect [ADE]: 0.64, *p* < 2 × 10^−16^, Figure 6d). While the trending reduction in muscle mass exerted a minor counteracting indirect effect (Average Causal Mediation Effect [ACME]: −0.194, *p* = 0.004), the combined outcome was a significant net increase in contractile force driven by CN19o (Total Effect: 0.45, *p* < 2 × 10^−16^). H&E staining showed a similar gross appearance of CN19o‐expressing muscle fibers to EGFP controls, with no signs of endomysial inflammation or necrotic fibers in either condition (Figure 6e). The minimum Feret diameters of individual CN19o‐expressing muscles were not significantly different from contralateral EGFP‐expressing controls (Figure 6f, *p* = 0.2437). These observations suggest that short‐term CN19o expression improves contractility without significantly altering muscle mass or fiber size.

To exclude the possibility that the apparent benefit of CN19o was merely an artifact of EGFP‐induced force suppression in the contralateral control muscles, a separate cohort of 22‐month‐old mice received AAV9‐tMCK‐CN19o in one TA and PBS in the contralateral TA. Four months later, CN19o‐expressing muscles again generated significantly greater absolute force (linear mixed‐effect model, estimate = 0.5 [0.11, 0.89], *p* = 0.0131, Figure 6g) and normalized force (linear mixed‐effect model, estimate = 0.96 [0.33, 1.59], *p* = 0.0033, Figure 6i) despite a modest decrease in muscle mass (~7%, *p* = 0.0163; Figure 6h). Mediation analysis confirmed a robust direct effect of CN19o on force (ADE: 0.81, *p* = 0.002) that was only partially offset by reduced mass (ACME: −0.32, *p* = 0.038), yielding a significant net force increase (Total effect: 0.49, *p* = 0.016; Figure 6j).

## 3 Discussion

Aging disrupts Ca^2+^ and redox homeostasis in skeletal muscle, with oxidative damage, RyR1 oxidative/nitrosative remodeling, SR Ca^2+^ leak, impaired tetanic Ca^2+^ release, and defective activity‐coupled mitochondrial Ca^2+^ signaling (Mecocci et al. 1999; Andersson et al. 2011; Lamboley et al. 2016; Pietrangelo et al. 2015). These abnormalities impair excitation–contraction and excitation–metabolism coupling, contributing to intrinsic muscle weakness. Yet Ca^2+^ and redox signaling are also essential for muscle performance and exercise adaptation, making their effects context‐dependent (Egan and Sharples 2023; Powers et al. 2024). As a Ca^2+^/calmodulin‐ and redox‐regulated kinase, CaMKII is positioned to transduce both physiological contractile signals and age‐associated Ca^2+^ and redox dysregulation. In young skeletal muscle, CaMKII supports acute contractile performance and exercise‐responsive transcriptional adaptation (Chin 2005; Tavi and Westerblad 2011; Wang et al. 2021; Tsukahara et al. 2026).

Here, we show that CaMKII abundance is increased in aged mouse skeletal muscle and that sustained CaMKII activation in young muscle is sufficient to impair contractile function, disrupt mitochondrial localization, and shift the transcriptome toward an aged profile. Conversely, expression of the CaMKII inhibitory peptide CN19o in aged muscle improves contractile force without inducing hypertrophy. NF‐κB blockade did not prevent the initial CaMKII^CA^‐induced force deficit but partially attenuated its long‐term progression, indicating that additional downstream mechanisms are involved. Together, these findings identify sustained CaMKII signaling as a contributor to age‐associated muscle dysfunction within the multifactorial pathogenesis of sarcopenia. This context‐dependent shift in CaMKII function from adaptive signaling in youth to maladaptive signaling in aging supports CaMKII as an example of antagonistic pleiotropy (Rose 1994).

We found that total CaMKII abundance increased in aged mouse skeletal muscle, whereas T287 autophosphorylation varied across age, muscle group, and isoform. In our RNA‐seq dataset, *Camk2b* was significantly increased with aging, whereas *Camk2g* and *Camk2d* were not, suggesting that increased CaMKIIβ protein abundance may partly reflect transcriptional upregulation, while increased CaMKIIδ/γ protein abundance likely reflects post‐transcriptional regulation. T287 autophosphorylation is an important marker of CaMKII activity but captures only one regulatory state of a kinase also controlled by Ca^2+^/calmodulin binding, redox‐dependent modification, subcellular localization, and substrate availability (Brown and Bayer 2024). Thus, increased CaMKII abundance may amplify signaling driven by age‐associated Ca^2+^ and redox dysregulation. These observations motivated us to test whether sustained CaMKII activation is sufficient to impair muscle function and whether CaMKII inhibition can improve contractile function in aged muscle.

To model sustained CaMKII signaling in skeletal muscle, we used intramuscular AAV9 delivery to express a constitutively active CaMKIIγ (CaMKIIγ‐T287D/CaMKII^CA^) under the control of the muscle‐specific tMCK promoter in young muscle. CaMKII^CA^ expression reduced endogenous CaMKII abundance and T287 autophosphorylation, potentially reflecting feedback regulation, as active CaMKII conformations can promote ubiquitin‐dependent CaMKII degradation (Quasney and Griffith 2025). Despite this reduction, CaMKII^CA^ increased overall CaMKII activity, as demonstrated by kinase activity assays in muscle lysates and the CaMKII activity reporter CaMKAR in isolated muscle fibers.

Sustained CaMKII activation reduced muscle force before atrophy became a major contributor to weakness. At 6 weeks after CaMKII^CA^ expression, force was reduced across the stimulation‐frequency range and remained lower after normalization to muscle mass. Mediation analysis further indicated that this early contractile defect was independent of muscle mass loss. By 9 months, however, substantial atrophy had developed and accounted for a major component of the force deficit. Thus, sustained CaMKII signaling initially impairs muscle quality and, with prolonged activation, contributes to progressive atrophy. This temporal pattern resembles human muscle aging, in which loss of strength often precedes or exceeds loss of muscle mass (Sayer and Cruz‐Jentoft 2022). Consistent with our findings, intramuscular AAV1 expression of constitutively active CaMKIIβ under the CMV promoter was previously shown to reduce muscle mass and force in young mice over 3 months (Eguchi and Yamanashi 2022). Our muscle‐specific model extends these findings by confirming increased CaMKII activity, defining the functional time course, and linking sustained CaMKII signaling to mitochondrial spatial remodeling and aging‐associated transcriptional changes.

A prominent structural consequence of CaMKII^CA^ expression was disrupted mitochondrial localization without evidence of generalized mitochondrial depletion. CaMKII^CA^‐expressing muscles showed subsarcolemmal and intermyofibrillar mitochondrial aggregation by COX and SDH staining and electron microscopy, together with reduced Z‐line mitochondrial occupancy at both early and later time points. In contrast, the mitochondrial‐to‐nuclear DNA ratio and the abundance of representative respiratory‐chain subunits were not significantly reduced, indicating altered mitochondrial organization rather than loss of mitochondrial content. In adult skeletal muscle, intermyofibrillar mitochondria are normally positioned near I‐bands adjacent to Z‐lines and closely associated with Ca^2+^ release units (CRUs), supporting local ATP delivery, SR Ca^2+^ reuptake, and activity‐coupled mitochondrial Ca^2+^ uptake (Boncompagni et al. 2009; Ainbinder et al. 2015). Aging similarly disrupts this organization, with reduced CRU–mitochondria apposition, fewer structural tethers, mitochondrial displacement, and impaired activity‐coupled mitochondrial Ca^2+^ signaling in aged mouse muscle (Pietrangelo et al. 2015). Altered mitochondrial organization is also observed in aged human skeletal muscle (Scudese et al. 2025).

CaMKII^CA^‐induced mitochondrial mislocalization could impair contractile function through at least two non‐mutually exclusive mechanisms. Displacement from CRU‐associated domains may disrupt excitation–metabolism coupling by reducing mitochondrial Ca^2+^ uptake during muscle activity (Ainbinder et al. 2015; Pietrangelo et al. 2015). Misalignment with the contractile apparatus may also limit local ATP supply for cross‐bridge cycling and Ca^2+^ reuptake (Milner et al. 2000; Winter et al. 2015). Thus, mitochondrial mislocalization represents one plausible contributor to CaMKII^CA^‐induced and aging‐associated muscle dysfunction.

How CaMKII^CA^ disrupts mitochondrial organization remains unclear. One possibility is altered mitochondrial dynamics, as CaMKII can phosphorylate and activate the mitochondrial fission regulator Drp1 (Xu et al. 2016). Another is disruption of cytoskeletal anchoring, as loss of desmin or plectin produces mitochondrial aggregation resembling that observed in CaMKII^CA^‐expressing muscle (Milner et al. 2000; Winter et al. 2015). CaMKII can phosphorylate desmin and other non‐epithelial intermediate filament proteins in vitro (Tokui et al. 1990). Sustained CaMKII signaling may therefore disrupt mitochondrial localization through altered mitochondrial dynamics, cytoskeletal anchoring, or both.

Beyond these structural changes, sustained CaMKII activation also produced broad transcriptional remodeling. RNA‐seq analysis showed that young CaMKII^CA^‐expressing muscles acquired a transcriptomic profile resembling that of aged muscle. GSEA identified pathways similarly altered by CaMKII activation and aging, including immune activation, DNA damage response, mRNA splicing, growth and death signaling, and extracellular matrix remodeling. Notably, CN19o expression in aged muscle produced a subtle global shift opposite to age‐associated transcriptional changes, indicating that CaMKII contributes to, but does not fully account for, the aging‐associated gene‐expression program. Consistent with this interpretation, CaMKII inhibition improved contractile function without increasing muscle mass. Although chronic inflammation contributes to sarcopenia and CaMKII can activate NF‐κB (Martin et al. 2018; Granic et al. 2023), ISR‐mediated blockade of canonical NF‐κB signaling did not improve the early CaMKII^CA^‐induced force deficit but partially preserved force after prolonged CaMKII activation without preserving muscle mass. Thus, canonical NF‐κB signaling contributes to the progressive contractile decline induced by sustained CaMKII activation but does not account for the early force deficit or muscle atrophy, implicating additional downstream pathways.

Mediation analysis identified the heme metabolism pathway as a mediator of CaMKII^CA^‐induced force decline. Consistent with the close coupling of heme and iron homeostasis (Dutt et al. 2022; Galy et al. 2024), CaMKII^CA^ altered transcripts involved in heme synthesis and export, mitochondrial heme handling, iron transport, and iron storage. CollecTRI analysis further identified Spic as the top‐ranked activated transcription factor in CaMKII^CA^‐expressing young muscle and the top‐ranked suppressed transcription factor in CN19o‐expressing aged muscle. Spic is induced by heme‐dependent degradation of Bach1 and promotes the differentiation of iron‐recycling macrophages (Haldar et al. 2014). Thus, reciprocal Spic activity inferred from these datasets is consistent with the heme/iron‐associated transcriptional signature. However, Spic activity was inferred from bulk‐muscle RNA‐seq; we cannot determine whether this transcriptional feature originates from myofibers or muscle‐associated immune cells, nor does the association establish Spic as a direct downstream target of CaMKII.

These coordinated changes suggest dysregulated heme/iron handling driven by sustained CaMKII activity, but do not define the underlying biochemical state. One possible model is functional iron deficiency coexisting with labile‐iron accumulation. Impaired incorporation of iron into heme and iron–sulfur clusters could compromise mitochondrial metabolism and other iron‐dependent processes, while expansion of the labile‐iron pool could increase oxidative stress. Iron dyshomeostasis has been reported in aging human skeletal muscle and has been associated with mitochondrial DNA damage (Picca et al. 2019). The stress‐response and DNA‐repair signatures in CaMKII^CA^‐expressing muscle are compatible with, but not specific to, this model. Sustained CaMKII activity could therefore link age‐associated Ca^2+^/redox dysregulation to iron dyshomeostasis. Direct measurements of heme, labile and total iron, and heme‐ and iron–sulfur cluster‐dependent function will be required to test this model.

Similar context dependence occurs in other major signaling pathways that regulate skeletal muscle mass and function. Although mTORC1 promotes anabolic growth, excessive mTORC1 signaling has been implicated in age‐related muscle loss and dysfunction (Joseph et al. 2019). In contrast, Akt signaling appears to remain protective, as its activation promotes hypertrophy and force production, whereas skeletal muscle Akt1/2 deficiency accelerates sarcopenia‐like muscle loss and functional decline (Blaauw et al. 2009; Sasako et al. 2022). Together with our findings on CaMKII, these observations illustrate that the effects of signaling pathways in aging depend not simply on their canonical physiological functions, but on pathway‐specific biology as well as the magnitude, duration, and cellular context of activation.

We propose that exercise‐induced and aging‐associated CaMKII activation arise from disturbances in cellular homeostasis (Figure 6k). In young muscle, exercise induces transient CaMKII activation that scales with physiological demand and resolves during recovery (Rose and Hargreaves 2003; Egan et al. 2010). With aging and declining functional capacity, however, everyday activity may impose greater relative physiological stress, promoting more persistent CaMKII activation and shifting its effects toward dysfunction. Exercise may therefore be protective not only by inducing beneficial adaptation but also by preserving functional reserve and reducing chronic homeostatic stress.

For individuals unable to achieve sufficient benefit from exercise, CaMKII inhibition may represent a complementary strategy. The FDA‐approved JAK inhibitor ruxolitinib was recently identified as a potent CaMKII inhibitor at therapeutic concentrations (Reyes Gaido et al. 2023). Ruxolitinib improved physical function in naturally aged mice and has been associated with increased muscle mass in patients treated for myelofibrosis (Xu et al. 2015; Lucijanic et al. 2021). Although these effects have been attributed primarily to JAK inhibition, the potential contribution of CaMKII inhibition warrants investigation. Together, our findings identify sustained CaMKII signaling as a modifiable contributor to age‐related loss of muscle quality and provide a framework for distinguishing physiological from maladaptive CaMKII activation in aging muscle.

### 3.1 Limitations

This study has several limitations. First, constitutively active CaMKIIγ models sustained CaMKII activation but do not reproduce the endogenous magnitude, duration, isoform composition, or subcellular distribution of CaMKII dysregulation in aging muscle. Second, although mitochondrial mislocalization, aging‐associated transcriptional changes, and the heme/iron pathway were linked to CaMKII^CA^‐induced force loss, their causal contributions remain to be established experimentally. Finally, CN19o improved contractile function in aged muscle, but the endogenous CaMKII isoforms and subcellular pools responsible for this effect remain unresolved, and the relevance of CaMKII inhibition to human muscle aging requires further investigation.

## 4 Experimental Procedures

A detailed description of experimental procedures is in the Supporting Information.

## Author Contributions

Q.W. conceptualized the study. Q.W. and M.R.B. wrote the manuscript. Q.W. and P.M.A. carried out project administration and supervision. Q.W., M.R.B., T.C., E.H.‐O., and P.M.A. designed experiments. M.R.B., Q.W., T.C., G.R.‐S., E.H.‐O., C.A., L.F., A.L., Q.‐L.X., E.E., and A.H. analyzed data. M.R.B., Q.W., E.D.L., G.L.‐C., A.L., W.A.F., G.R.‐S., and S.J.J. performed experiments. All authors discussed and edited the manuscript.

## Funding

M.R.B. is supported by the National Institute on Aging (2T32AG058527‐06A1) and the National Institute of Arthritis and Musculoskeletal and Skin Diseases (5T32AR048522‐20). Q.W. is supported by the Glenn Foundation for Medical Research and AFAR Grants for Junior Faculty, Karen and Ethan Leder CIM Human Aging Project Scholarship, Nathan Shock Scholarship, the Johns Hopkins University Claude D. Pepper Older Americans Independence Center, which is funded by the National Institute on Aging of the National Institutes of Health (NIH) under award number P30AG021334 and NIH grant K24AG088484 (P.M.A.); and the Johns Hopkins Center for AIDS Research Faculty Development Grant P30AI094189. E.H.‐O. was supported by the National Institutes of Health grant R01‐AR075726. L.F. is supported by NIH award R35GM156374.

## Conflicts of Interest

The authors declare no conflicts of interest.

## Supporting information


**Figure S1:** Age‐associated changes in muscle mass, CaMKII abundance, and CaMKII autophosphorylation across skeletal muscles. (a) Gastrocnemius and quadriceps muscle mass normalized to body weight in young (3.7‐month‐old, *n* = 10, 5 male/5 female), old (20‐month‐old, *n* = 10, 5 male/5 female), and very old (33‐month‐old, *n* = 21, 21 male) mice. Each point represents one mouse. Data were analyzed by Welch's ANOVA followed by Dunnett's T3 multiple‐comparisons test. Exact *p* values are shown in the graphs. (b) Representative Western blots of pT287‐CaMKII, total CaMKII, and total protein stain in young (3.7‐month‐old, *n* = 10; 5 male/5 female) and old (20‐month‐old, *n* = 10; 5 male/5 female) TA muscles. Quantification of pT287‐CaMKIIβ, pT287‐CaMKIIδ/γ, total CaMKIIβ, and total CaMKIIδ/γ normalized to total protein stain is shown. Each point represents one mouse. Data were analyzed by Student's *t*‐test; *p* values are shown in the graphs. (c) Representative Western blots of pT287‐CaMKII, total CaMKII, and total protein stain in young (3.7‐month‐old, *n* = 10; 5 male/5 female) and very old (33‐month‐old, *n* = 14; 14 male) gastrocnemius muscles. Quantification of pT287‐CaMKIIβ, pT287‐CaMKIIδ/γ, total CaMKIIβ, and total CaMKIIδ/γ normalized to total protein stain is shown. Each point represents one mouse. Data were analyzed by Student's *t*‐test; *p* values are shown in the graphs. Data are mean ± SEM.
**Figure S2:** Chronic CaMKII activation further impairs muscle contractility, muscle mass, and histological structure. (a) In vivo isometric force‐frequency curves of TA muscles approximately 9 months after injection with PBS in one TA and AAV9‐tMCK‐CaMKIICA in the contralateral TA. *n* = 6 mice (3 male/3 female; 6 muscles per condition). Data were analyzed by linear mixed‐effects model; estimate = −0.99, 95% CI [−1.39, −0.59], *p* = 4.21 × 10^−6^. (b) TA muscle mass approximately 9 months post‐injection. *n* = 6 mice (3 male/3 female). Data were analyzed by paired Student's *t*‐test; the *p* value is shown in the graph. The animals contributing to panels (a) and (b) were not identical: out of seven mice in this cohort, one animal underwent contractility testing but was lost before tissue collection, whereas another was sacrificed and contributed muscle‐mass measurements before contractility testing. (c) Isometric force normalized to TA muscle mass. This analysis included the 5 mice with both contractility and muscle‐mass measurements (3 male/2 female; 5 muscles per condition). Data were analyzed by linear mixed‐effects model; estimate −0.99, 95% CI [−1.39, −0.59], *p* = 4.21 × 10^−6^. (d) Mediation analysis using the R mediation package. (e) Representative H&E, modified Gomori's trichrome (GT), and COX staining of TA cross‐sections approximately 9 months post‐injection from PBS‐ and AAV9‐tMCK‐CaMKIICA‐injected paired muscles. (f) Muscle fiber minimum Feret diameter from 4 paired mice (1 male/3 female), approximately 9 months post‐injection. Each point represents the median of approximately 200–300 fibers from one muscle. *n* = 4 muscles per treatment. Data were analyzed by paired Student's *t*‐test; the *p* value is shown in the graph. (g) Distribution of individual muscle fiber minimum Feret diameters pooled across the 4 muscles per treatment shown in panel (f).
**Figure S3:** Short‐term CaMKIICA expression does not alter mitochondrial complex abundance or mtDNA copy number in TA muscles 7 weeks post‐injection. (a) Western blot detection of representative subunits of mitochondrial complexes I–V. (+) denotes AAV9‐CaMKIICA‐injected TA, and (−) denotes AAV9‐EGFP‐injected TA. (b) Quantification of individual subunit expression levels relative to total protein staining. *n* = 7 EGFP‐expressing muscles and *n* = 5 CaMKIICA‐expressing muscles. Samples were from six mice: five mice (3 male, 2 female) expressed EGFP and CaMKIICA in contralateral TA muscles, and one female mouse expressed EGFP in both TA muscles; multiple *t*‐tests, nd = not a discovery (not significant). (c) Quantification of mitochondrial DNA to nuclear DNA copy number ratio by qPCR. *n* = 9 paired mice (4 male/5 female). Data were log‐transformed and analyzed by paired Student's *t*‐test; *p* value is shown in the graph.
**Figure S4:** Long‐term CaMKIICA expression does not change the abundance of mitochondrial complexes I‐V in TA muscles approximately 9 months post‐injection. (a) Western blot detection of representative subunits of mitochondrial complexes I–V. (+) denotes AAV9‐tMCK‐CaMKIICA‐injected TA, and (−) denotes PBS‐injected contralateral TA. (b) Quantification of individual subunit expression levels. *n* = 5 paired mice (2 male/3 female). Data were analyzed by paired multiple *t*‐tests; nd, not a discovery (not significant).
**Figure S5:** CollecTRI regulatory network analysis. (a) Activated and repressed transcription factors by CaMKIICA expression (young CaMKIICA vs. young EGFP). (b) Activated and repressed transcription factors by CN19o expression (old CN19o vs. old EGFP).

**Table S1:** Reactome pathway enrichment associated with aging and CaMKII modulation in skeletal muscle. Gene set enrichment analysis of Reactome canonical pathways is summarized for old EGFP versus young EGFP muscle, young CaMKII^CA^ versus young EGFP muscle, and old CN19o versus old EGFP muscle. “pathway” and “pathway_clean” provide the original and simplified pathway names. “In_Age_Network” identifies pathways included in the aging network, while “Cluster” and “Cluster_Label” group related pathways and name each cluster; “representativeness” indicates how well a pathway represents its cluster. Normalized enrichment scores (NES), nominal *p* values (pval), FDR‐adjusted *p* values (padj), and significance indicators (Sig) are reported separately for aging, CaMKII^CA^, and CN19o. The “concordance” and “vs_Age” fields indicate whether the CaMKII^CA^‐ or CN19o‐associated pathway change occurs in the same or opposite direction as aging. “size” indicates the number of genes in the pathway.

**Table S2:** Hallmark gene sets and eigenpathway compositions used for mediation analysis. Hallmark gene‐set GSVA enrichment scores with |ρ| ≥ 0.8 were aggregated into two composite eigenpathways to reduce redundancy. Together with 13 individual Hallmark gene sets, these composites yielded 15 candidate variables evaluated as potential mediators of the effects of CaMKII^CA^ on muscle mass and contractile force.

**Data S1:** acel70704‐sup‐0004‐supinfo.docx.

**Data S2:** acel70704‐sup‐0005‐supinfo.xlsx.

**Data S3:** acel70704‐sup‐0006‐supinfo.xlsx.

## Data Availability

The data that support the findings of this study are available in the Supporting Information material of this article and are available in the open data repository Zenodo.org (10.5281/zenodo.22035913).
