# Supplementary material for: Sustained Activation of CaMKII Promotes Skeletal Muscle Contractile Dysfunction in Aging
Source: Aging Cell. 2026 Sep 10;25(9):e70704. doi: 10.1111/acel.70704 (PMC13560506; doi:10.1111/acel.70704)
Supplement: Supplementary file 1 [file ACEL-25-e70704-s002.docx]

**SUPPLEMENTARY MATERIALS**

**Full EXPERIMENTAL PROCEDURES**

4.1 Animals

An equal number of male and female C57BL/6JN mice were obtained from the National Institute on Aging (NIA) Aged Rodent Colony. All animal procedures adhered to National Institutes of Health (NIH) guidelines and were approved by the Johns Hopkins University School of Medicine Institutional Animal Care and Use Committee. Mice were housed in the Johns Hopkins animal facility under a 12-hour light/dark cycle and provided 2018 Teklad Global 18% Protein Rodent Diet ad libitum. The housing environment was maintained at 22 ± 1°C with a humidity level of 40 ± 10%.

4.2 Western blotting

Muscle samples were homogenized in T-PER™ reagent (Thermo Fisher Scientific, #78510) containing cOmplete™ Protease Inhibitor Cocktail (Roche, #11697498001) and PhosSTOP™ Phosphatase Inhibitor Cocktail (Roche, #4906845001) using a Precellys homogenizer. After centrifugation at 10,000 × g for 10 minutes, 5 μg (for mitochondrial proteins) or 20 μg (for all other proteins) of protein from supernatants was resolved on NuPAGE Tris-Bis gels and transferred to PVDF membranes. Total protein was visualized using Pierce Reversible Protein Stain (Thermo Fisher Scientific, 24585) before membranes were blocked with EveryBlot buffer (Bio-Rad, 12010020) for 5 minutes at room temperature. Membranes were incubated with primary antibodies at optimized dilutions, followed by HRP-conjugated secondary antibodies (20 ng/mL). Immunoreactive bands were detected using SuperSignal™ West Dura Substrate (Thermo Fisher Scientific) on a Bio-Rad Gel Doc XRS+ system and analyzed with Image Lab software (v6.1), with normalization to total protein content as determined by reversible staining.

Primary antibodies: total-CaMKII (BD biosciences, 611292, 1:3000), pT287-CaMKII (Cell Signaling Technology, D21E4, 1:2000), FLAG (Thermo Fisher Scientific, MA1-91878, 1:2000), EGFP (Abcam, ab290, 1:2000), OxPhos Rodent WB Antibody Cocktail (Thermo Fisher Scientific, 458099, 1:1000).

4.3 AAV9 viral vector construction and injection

The AAV9-tMCK-CaMKII^CA^ construct was generated by appending a triple-FLAG tag (MDYKDHDGDYKDHDIDYKDDDDK) and a flexible linker (GGGGS) to the N-terminus of mouse CaMK2g (NM_001039138) coding sequence. To create the constitutively active CaMKII^CA^, the threonine residue at position 287 was mutated to aspartic acid (T287D) through codon substitution to GAC. This sequence was subcloned into a pAAV-tMCK-WPRE vector backbone, and the recombinant AAV9-tMCK-CaMKII^CA^ viral particles were produced by Vector BioLabs (Great Valley Parkway, Malvern PA).

AAV9-tMCK-CN19o was developed using a synthetic DNA sequence (ATGTACCCTTACGACGTACCGGACTACGCTGGCGGGGGAGGGAGTAAGCGGGCTCCAAAACTCGGCCAGATCGGAAGACAGAAAGCCGTGGATATTGAAGATTGA) encoding an HA tag followed by a flexible linker (GGGGS) and the CN19o peptide (KRAPKLGQIGRQKAVDIED). This construct was similarly cloned into the pAAV-tMCK-WPRE vector backbone, with viral packaging performed by Vector BioLabs.

The IκB super repressor (ISR) virus, AAV9-tMCK-ISR, was generated by introducing S32A and S36A mutations into the mouse Nfkbia coding sequence (NM_010907.2). The modified cDNA was then cloned into the pAAV[Exp]-tMCK-WPRE vector and packaged into AAV9 by VectorBuilder (Chicago, IL).

The control virus AAV9-tMCK-EGFP (SKU VB5037) was obtained from Vector BioLabs.

The viral constructs were diluted in sterile phosphate-buffered saline (PBS) to a final concentration of 2.0 × 10^12^ viral genomes/mL and administered using 31-gauge needles. Each tibialis anterior (TA) muscle of anesthetized mice (2% isoflurane) received two 25 μL injections at separate sites, delivering a total dose of 1.0 × 10^11^ viral genomes per muscle (Cho et al. 2019). To minimize experimental bias, control and treatment viruses were alternated between left and right legs across different animals, and the experimenter conducting the muscle contractility assay was blinded to the identity of the injected viruses.

4.4 Single fiber CaMKAR imaging

Isolated muscle fiber CaMKAR imaging from skeletal muscle injected with AAV9-tMCK-CaMKII^CA^.

All the animal procedures and protocols were reviewed and approved by the Institutional Animal Care and Use Committees of the University of Maryland. Male C57BL/6J mice (Charles River, Wilmington, MA) were used. All mice used were 8–12 weeks of age. Environmental conditions were maintained with a 12-h light/dark cycle and constant temperature (21–23°C) and humidity (55 ± 10%). The cages contained corncob bedding (Harlan Teklad 7902) and environmental enrichment (cotton nestlet). Mice were supplied with dry chow (irradiated rodent diet; Harlan Teklad 2981) and allowed water ad libitum. C57BL/6 mice were infected at 8 weeks of age via intramuscular injection into the flexor digitorum brevis (FDB) muscle. A total dose of 1.0 × 10¹¹ viral genomes per muscle was administered. Subsequently, to facilitate the expression of the CaMKAR reporter system in vivo, electroporation was performed on the FDB muscles of 11-week-old mice (Banks et al. 2021). Subcutaneous injection of 20-30 μl of hyaluronidase (2.5 mg/ml) was administered into the plantar pad of a mouse anesthetized with 3% - 4.5% isoflurane in O₂ (1 L/min), using a 33-gauge needle. One hour later, the mouse was again anesthetized, and ~60-70 μg of CaMKAR plasmid DNA was injected into the footpad. The animal was kept on an isothermal pad for 5 minutes; then, two surgical stainless-steel electrodes were placed subcutaneously close to the proximal and distal tendons of the FDB muscle and 20 pulses of 100 V/cm, 20 ms in duration, were applied at 1 Hz via a commercial high current capacity output stage (ECM 830, BTX, Harvard Apparatus, Holliston, MA). One week later, single muscle fibers were enzymatically dissociated from the injected FDB muscles and cultured as described below (Bibollet et al. 2023). FDB muscle was isolated from male adult mice, enzymatically dissociated with collagenase type I (Sigma-Aldrich, St. Louis, MO) in spinner minimum essential medium (S-MEM; Cat. No. 11380-037; Gibco, Carlsbad, CA) with 10% FBS (Cat. #100–106; Gemini Bio-Products, West Sacramento, CA) for 3-4 hours at 37˚ C. Muscle was then gently triturated to separate fibers in S-MEM with FBS 10%. Fibers were plated in MEM culture media with 2% FBS on glass-bottomed dishes (MatTek Cor, Ashland, MA, Cat. No. P35G-1.0-14-C) coated with laminin (Thermo Fisher, Rockford, IL, Cat. No. 23017-015). Fibers were maintained in culture for 2 to 20 hours at 37 °C, 5% CO2 before the experiments. Positively transfected fibers were identified using an intensiometric approach by assessing the fluorescence expression profile excited with 488 nm and emitted light collected with a long-pass filter >510 nm. Cultured skeletal muscle FDB fibers expressing CaMKAR constructs were imaged in 2 mL of L-15 media (ionic composition in mM: 137 NaCl, 5.7 KCl, 1.26 CaCl2, 1.8 MgCl2, pH 7.4; Life Technologies, Carlsbad, CA). All single fiber recordings were performed at room temperature, 22°C. Individual muscle fibers expressing CaMKAR were excited with a 488 nm laser, and the fluorescence emitted >505 nm was acquired with an Olympus IX70 inverted microscope equipped with an Olympus FLUOVIEW 500 laser scanning confocal microscope imaging system using an Olympus 40X UPlanXApo objective. Fluorescence was calculated by the average of the intracellular fluorescent signal, considering three different regions of the isolated fibers (after subtracting the background extracellular signal). Five to seven single fibers per culture plate were analyzed independently for each animal. Regions of interest (ROIs) in the images were quantified using ImageJ software (National Institutes of Health, Bethesda, MD).

4.5 CaMKII Activity Assay

Tibialis anterior muscles were homogenized in lysis buffer (50mM Tris and 100mM NaCl) with protease and phosphate inhibitors (Sigma-Aldrich). CaMKII activity was measured using the SignaTECT® Protein Kinase Assay System (Promega, V8161) with ATP [γ-^32^P] (Revvity) according to the manufacturer’s instructions. Muscle lysates were incubated with the biotinylated peptide substrate in buffers containing either Ca^2+^ and calmodulin (calcium-activated activity) or EGTA (calcium-independent activity). 20 μL kinase reactions were incubated at 30°C for 10 minutes, after which 10 μL was spotted onto the Biotin Capture Membrane in duplicate and washed three times to remove unincorporated ATP. ^32^P incorporation was quantified by liquid scintillation counting. CaMKII activity was normalized to total protein concentration (Pierce™ BCA Protein Assay Kit, Thermo Fisher Scientific) and expressed as the ratio of Ca^2+^/calmodulin-independent to Ca^2+^/calmodulin-activated activity.

4.6 In vivo muscle contractility measurements

Muscle contractility was assessed using the Aurora 1300A system, as previously described (Westbrook et al. 2020). Briefly, mice were anesthetized with 4% isoflurane for induction and maintained at 1.5% during the procedure. They were positioned supine on a warming pad to sustain body temperature, with the knee stabilized and the foot secured to the footplate. Percutaneous stimulation of the peroneal nerve was applied using 100 μs pulses, with current optimized to elicit maximal isometric force. The force-frequency relationship was assessed using 250 ms pulse trains ranging from 1 to 150 Hz. Data were exported from DMA-HT analysis software (Aurora Scientific), analyzed in R, and visualized using GraphPad Prism 10.

4.7 Histology

Whole TA muscles were frozen in liquid nitrogen-cooled isopentane and sectioned into 8 μm sections. The samples were stained with the Hematoxylin and Eosin staining kit (Vector Laboratories, H-3502). Protocols for modified Gomori’s trichrome staining, SDH, and COX staining were obtained from the Washington University Neuromuscular Disease Center.

For TEM, samples were fixed in 2.5% glutaraldehyde, 3 mM MgCl_2_ in 0.1 M sodium cacodylate buffer, pH 7.2, overnight at 4 °C. After buffer rinse, samples were postfixed in 2% osmium tetroxide in 0.1 M sodium cacodylate for at least one hour (no more than two) on ice in the dark and samples rinsed in distilled water, dehydrated in a graded series of ethanol and embedded in Epon (PolySci) resin. Samples were polymerized at 60 °C overnight.

Thin sections, 60 to 90 nm, were cut with a diamond knife on a Leica UCT ultramicrotome and picked up with 2x1 mm Formvar copper slot grids. Grids were stained with 3% uranyl acetate (aq.) followed by lead citrate and observed with a Hitachi 7600 TEM at 80 kV. Images were captured with an AMT CCD XR80 (8-megapixel camera - side mount AMT XR80 – high-resolution high-speed camera).

Light and TEM images were analyzed in Fiji ImageJ with treatments blinded to the analyzer (Schindelin et al. 2012). For light microscopy, two fields were imaged from a single mid-belly cross-section of each muscle sample, with one field from the superficial region and one from the deep region. Muscle fiber size was quantified from H&E-stained TA cross-sections. Muscle fibers were manually outlined with the freehand selections tool, and the minimum Feret diameter was measured for each outlined fiber. Minimum Feret diameter, also termed lesser diameter, was used as a robust index of muscle fiber size that is less affected by sectioning angle (Dubowitz et al. 2020). Approximately 200-300 fibers were measured per muscle (Dubowitz et al. 2020). For statistical analysis, the median minimum Feret diameter from each muscle was treated as one biological replicate, and paired EGFP- and CaMKII^CA^-expressing muscles from the same animal were compared by paired Student’s t-test. Raw histology images are available upon request.

TEM micrographs were acquired by a microscopy core facility operator blinded to treatment groups. To quantify intermyofibrillar mitochondrial localization relative to Z-lines, 3-7 longitudinal TEM images acquired at 8500x magnification were analyzed per muscle, depending on image availability and section quality. Clearly identifiable Z-lines were scored according to mitochondrial presence at the two ends of the Z-line: 1 when mitochondria were present at both ends, 0.5 when mitochondria were present at one end, and 0 when mitochondria were absent from both ends. For each muscle, the Z-line mitochondrial occupancy score was calculated as the mean score across all scored Z-lines from all analyzed micrographs. Paired EGFP- and CaMKII^CA^-expressing muscles were compared by paired Student’s t-test.

4.8 Mitochondrial DNA copy number quantification

Total DNA was isolated from tibialis anterior muscle using the DNeasy Blood & Tissue Kit (QIAGEN) following Proteinase K digestion at 56°C and normalized to 20 ng/μL. Relative mitochondrial DNA (mtDNA) copy number was quantified by qPCR using PowerTrack SYBR Green Master Mix (Thermo Fisher Scientific), with 40 ng DNA and 0.4 μM of each primer in a 20 μL reaction. Samples were analyzed in duplicate with a no-template control included on each plate, using a fast amplification protocol followed by melt-curve analysis. The mtDNA-specific primers mMitoF1 (5′-CTAGAAACCCCGAAACCAAA-3′) and mMitoR1 (5′-CCAGCTATCACCAAGCTCGT-3′), and nuclear DNA primers targeting B2m, mB2MF1 (5′-ATGGGAAGCCGAACATACTG-3′) and mB2MR1 (5′-CAGTCTCAGTGGGGGTGAAT-3′), were described by (Malik et al. 2016) and designed to quantify mtDNA without co-amplification of nuclear mitochondrial insertion sequences. Relative mtDNA content was expressed as the mitochondrial-to-nuclear DNA ratio.

4.9 RNA sequencing and analyses

Total RNA was isolated from TA muscle samples five weeks after viral injection using the Direct-zol RNA MiniPrep Kit (Zymo Research, R2050). RNA quality was assessed with a Fragment Analyzer (Advanced Analytical Technologies, Inc.), and all samples had an RNA Quality Number (RQN) >7.0. mRNA enrichment was performed using 1 μg of total RNA per sample with the NEBNext® Poly(A) mRNA Magnetic Isolation Module (New England Biolabs, E7490L), followed by RNA library preparation with the NEBNext® Ultra^TM^ II Directional RNA Library Prep Kit (New England Biolabs, E7765L). Each library was uniquely barcoded using the NEBNext Multiplex Oligos for Illumina (96 Unique Dual Index Primer Pairs, New England Biolabs, E6440S). The RNA libraries were then submitted to the Johns Hopkins Genetic Resources Core Facility and sequenced on a NovaSeq X Plus (Illumina, Inc.) to generate 2 × 150 bp paired-end reads.

Transcriptomic data collected by RNA sequencing (RNA-seq) was analyzed to determine the genes that are present in each sample and condition, their expression levels, and the differences between expression levels among experiment conditions, as follows. Sequencing reads were mapped to the mouse genome version GRCm39 with the spliced alignment tool STAR 2.7.10a (Dobin et al. 2013). The aligned reads were assembled with PsiCLASS v.1.0.3 (Song et al. 2019) to create gene and transcript models. Transcripts were then assigned to known genes from the GENCODE v.M28 reference gene set augmented with the three transgene sequences (EGFP, CN19o, CaMKII^CA^) as additional “chromosomes”. Lastly, DESeq2 (Anders & Huber 2010) was used to quantify the expression levels and determine differentially expressed genes. Additional visualizations, including volcano plots and plots of principal component analysis (PCA) components, were visualized with custom R scripts (R v.4.1.3).

To account for similarities as the sequence level between the CaMKII^CA^ transgene and the endogenous *Camk2g* mouse gene, which contribute to multi-mapped reads that can alter the expression calculations for the two genes, we performed two analyses. The first analysis used the PsiCLASS gene annotations as created above. The second analysis identified and distinguished ‘shared’ versus ‘unique’ areas of the two genes, with the rationale that unique areas will provide a more accurate assessment of the genes’ expression levels. For the *Camk2g* gene, we separated the ‘unique’ 3’ most exon (chr14:20784942-20786920; internal gene ID ‘chr14.111872.e1’) from the rest of the gene, which shows sequence similarity to the transgene. For the CaMKII^CA^ transgene, we separated the first 200 bp (internal gene ID: ‘chr_CaMKIICA.402115’), which includes the 84 bp linker sequence, as ‘unique’, from the rest of the gene. These unique gene portions can be used to more accurately assess and compare the expression levels of these two genes. All the other genes’ expression levels are unaffected by the change in annotation.

Pathway networks were generated from the differential-expression results for the age, CaMKII^CA^, and CN19o contrasts. For each contrast, gene set enrichment analysis was performed using the fgsea implementation (Subramanian et al. 2005; Korotkevich et al. 2021) against Reactome pathway gene sets (Milacic et al. 2024) with pathway-size limits of 15–500 genes and 10,000 permutations; pathways with FDR-adjusted P < 0.05 for age were retained for network construction. Each retained Reactome pathway was represented as a network node, with node color reflecting the normalized enrichment score (NES) and node size reflecting pathway gene-set size. Pairwise pathway similarity was calculated using the Jaccard index based on the overlap of Reactome member genes, and edges were drawn between pathways with similarity ≥ 0.08. Pathways were clustered by hierarchical clustering of the Jaccard-derived dissimilarity matrix using Ward's method, with an adaptive number of initial clusters scaled to the number of significant pathways and subsequent merging of highly similar clusters using a mean inter-cluster similarity threshold of 0.10. Cluster positions were determined from the inter-cluster dissimilarity structure using multidimensional scaling, and individual pathway nodes were placed around their cluster centers with reproducible random jitter. Clusters were labeled by the representative pathway with the greatest within-cluster and least between-cluster gene sharing, and up- and down-enriched pathways were visualized with opposing color scales to generate the CaMKII^CA^ and CN19o transcriptome-derived Reactome pathway networks.

Correlation analysis was performed in R using the "cor.test" function. For each comparison at the transcript level, transcripts that were significant by P < 0.05 in either condition were included. For visualization purposes, BH-corrected Fisher's combined P values were used for bubble size.

Log₂-transformed expression matrices were subjected to gene-set variation analysis (GSVA) in R ("GSVA" version 1.48.0, "GSEABase" version 1.60.0) using the "Hallmark" gene sets. Partial Spearman correlations controlling for subject ID were computed with pcor.test ("ppcor" version 1.1), and an igraph network was constructed from edges with |ρ| ≥ 0.8; each connected component containing > 1 variable was collapsed to its first principal component ("stats" version 4.4.1), termed an "eigenpathway."

Transcription factor activity was inferred using the CollecTRI regulatory network retrieved via the R package OmnipathR and analyzed with decoupleR. Activities were estimated from the expression matrix using weighted-mean, univariate linear model, and VIPER-based enrichment methods."

4.10 Contractile Force Analysis

To assess changes in contractile force between conditions, linear mixed-effects models were generated in R using the “lmer” function of the “lme4” package (version 1.1.35.5). The “boxcox” function of the “MASS” package (version 7.3.61) was used to transform the force readings to ensure that the assumption of linearity and normality was met. Additionally, frequency values were log-transformed to ensure linearity, resulting in a final model of: BoxCoxForce ~ Condition+log(Frequency)+(1|ID), with ID representing random effects of subject to account for within-subject and repeated measures design.

4.11 Statistics

Statistical analyses were performed in GraphPad Prism 11 (GraphPad Software) and R version 4.4. The choice of test was determined by the structure and distribution of each dataset. For within-animal contralateral designs, paired analyses were used whenever both limbs were available; when a paired mate was missing, the corresponding unpaired test was used. Normality was assessed for each dataset, and parametric or non-parametric tests were selected accordingly. Two-tailed tests were used throughout.

Specifically, comparisons between two groups were made using paired or unpaired Student's t-tests when data were normally distributed, or the corresponding non-parametric test when they were not. Comparisons across more than two groups were made using Welch's ANOVA followed by Dunnett's T3 multiple-comparisons test. For force-frequency data, force readings were transformed using the Box-Cox procedure (boxcox function, MASS package, version 7.3.61) to satisfy the assumptions of linearity and normality prior to analysis.

Mediation Analysis: To determine the role of muscle mass in CaMKII^CA^- and CN19o-induced changes to contractile force, we employed mediation analysis using the “mediation” package (version 4.5.0). Mediator models were constructed as: muscle ~ Condition + (1|ID), and outcome models as: BoxCoxForce ~ Condition + muscle + log(Frequency) + (1|ID). For the construction of the combined SEM model (“lavaan” version 0.6.19), four mediators were included: heme metabolism, TGF-β signaling, protein secretion, and muscle mass, with sequential mediation paths from TGF-β signaling and protein secretion to muscle mass based on modification indices and biological plausibility.

Anders S & Huber W (2010) Differential expression analysis for sequence count data. *Nat Prec*, 1–1.

Banks Q, Bibollet H, Contreras M, Bennett DF, Bannister RA, Schneider MF & Hernández-Ochoa EO (2021) Voltage sensor movements of CaV1.1 during an action potential in skeletal muscle fibers. *PNAS* 118. Available at: https://www.pnas.org/content/118/40/e2026116118 [Accessed November 18, 2021].

Bibollet H, Bennett DF, Schneider MF & Hernández-Ochoa EO (2023) Functional Site-Directed Fluorometry in Native Cells to Study Skeletal Muscle Excitability. *JoVE*. Available at: https://app.jove.com/t/65311 [Accessed July 11, 2025].

Cho Y, Tachibana S, Hazen BC, Moresco JJ, Yates JR, Kok B, Saez E, Ross RS, Russell AP & Kralli A (2019) Perm1 regulates CaMKII activation and shapes skeletal muscle responses to endurance exercise training. *Mol Metab* 23, 88–97.

Dobin A, Davis CA, Schlesinger F, Drenkow J, Zaleski C, Jha S, Batut P, Chaisson M & Gingeras TR (2013) STAR: ultrafast universal RNA-seq aligner. *Bioinformatics* 29, 15–21.

Dubowitz V, Sewry CA & Oldfors A (2020) *Muscle Biopsy E-Book: A Practical Approach*, Elsevier Health Sciences.

Korotkevich G, Sukhov V, Budin N, Shpak B, Artyomov MN & Sergushichev A (2021) Fast gene set enrichment analysis. , 060012. Available at: https://www.biorxiv.org/content/10.1101/060012v3 [Accessed July 2, 2026].

Malik AN, Czajka A & Cunningham P (2016) Accurate quantification of mouse mitochondrial DNA without co-amplification of nuclear mitochondrial insertion sequences. *Mitochondrion* 29, 59–64.

Milacic M, Beavers D, Conley P, Gong C, Gillespie M, Griss J, Haw R, Jassal B, Matthews L, May B, Petryszak R, Ragueneau E, Rothfels K, Sevilla C, Shamovsky V, Stephan R, Tiwari K, Varusai T, Weiser J, Wright A, Wu G, Stein L, Hermjakob H & D’Eustachio P (2024) The Reactome Pathway Knowledgebase 2024. *Nucleic Acids Res* 52, D672–D678.

Schindelin J, Arganda-Carreras I, Frise E, Kaynig V, Longair M, Pietzsch T, Preibisch S, Rueden C, Saalfeld S, Schmid B, Tinevez J-Y, White DJ, Hartenstein V, Eliceiri K, Tomancak P & Cardona A (2012) Fiji: an open-source platform for biological-image analysis. *Nat Methods* 9, 676–682.

Song L, Sabunciyan S, Yang G & Florea L (2019) A multi-sample approach increases the accuracy of transcript assembly. *Nat Commun* 10, 5000.

Subramanian A, Tamayo P, Mootha VK, Mukherjee S, Ebert BL, Gillette MA, Paulovich A, Pomeroy SL, Golub TR, Lander ES & Mesirov JP (2005) Gene set enrichment analysis: A knowledge-based approach for interpreting genome-wide expression profiles. *Proceedings of the National Academy of Sciences* 102, 15545–15550.

Westbrook R, Chung T, Lovett J, Ward C, Joca H, Yang H, Khadeer M, Tian J, Xue Q-L, Le A, Ferrucci L, Moaddel R, Cabo R de, Hoke A, Walston J & Abadir PM (2020) Kynurenines link chronic inflammation to functional decline and physical frailty. *JCI Insight* 5. Available at: https://insight.jci.org/articles/view/136091 [Accessed October 3, 2022].
