# Supplementary material for: Sustained Activation of CaMKII Promotes Skeletal Muscle Contractile Dysfunction in Aging
Source: Aging Cell. 2026 Sep 10;25(9):e70704. doi: 10.1111/acel.70704 (PMC13560506; doi:10.1111/acel.70704)
Supplement: Supplementary file 2 [file ACEL-25-e70704-s006.docx]

SUPPLEMENTARY FIGURE 1 Age-associated changes in muscle mass, CaMKII abundance, and CaMKII autophosphorylation across skeletal muscles. (a) Gastrocnemius and quadriceps muscle mass normalized to body weight in young (3.7-month-old, n = 10, 5 male/5 female), old (20-month-old, n = 10, 5 male/5 female), and very old (33-month-old, n = 21, 21 male) mice. Each point represents one mouse. Data were analyzed by Welch's ANOVA followed by Dunnett's T3 multiple-comparisons test. Exact P values are shown in the graphs. (b) Representative Western blots of pT287-CaMKII, total CaMKII, and total protein stain in young (3.7-month-old, n = 10; 5 male/5 female) and old (20-month-old, n = 10; 5 male/5 female) TA muscles. Quantification of pT287-CaMKIIβ, pT287-CaMKIIδ/γ, total CaMKIIβ, and total CaMKIIδ/γ normalized to total protein stain is shown. Each point represents one mouse. Data were analyzed by Student’s t-test; P values are shown in the graphs. (c) Representative Western blots of pT287-CaMKII, total CaMKII, and total protein stain in young (3.7-month-old, n = 10; 5 male/5 female) and very old (33-month-old, n = 14; 14 male) gastrocnemius muscles. Quantification of pT287-CaMKIIβ, pT287-CaMKIIδ/γ, total CaMKIIβ, and total CaMKIIδ/γ normalized to total protein stain is shown. Each point represents one mouse. Data were analyzed by Student’s t-test; P values are shown in the graphs. Data are mean ± SEM.

SUPPLEMENTARY FIGURE 2 Chronic CaMKII activation further impairs muscle contractility, muscle mass, and histological structure. (a) In vivo isometric force-frequency curves of TA muscles approximately 9 months after injection with PBS in one TA and AAV9-tMCK-CaMKII^CA^ in the contralateral TA. n = 6 mice (3 male/3 female; 6 muscles per condition). Data were analyzed by linear mixed-effects model; estimate = -0.99, 95% CI [-1.39, -0.59], P = 4.21 × 10^-6^. (b) TA muscle mass approximately 9 months post-injection. n = 6 mice (3 male/3 female). Data were analyzed by paired Student’s t-test; the P value is shown in the graph. The animals contributing to panels (a) and (b) were not identical: out of seven mice in this cohort, one animal underwent contractility testing but was lost before tissue collection, whereas another was sacrificed and contributed muscle-mass measurements before contractility testing. (c) Isometric force normalized to TA muscle mass. This analysis included the 5 mice with both contractility and muscle-mass measurements (3 male/2 female; 5 muscles per condition). Data were analyzed by linear mixed-effects model; estimate -0.99, 95% CI [-1.39, -0.59], P = 4.21 × 10^-6^. (d) Mediation analysis using the R mediation package. (e) Representative H&E, modified Gomori’s trichrome (GT), and COX staining of TA cross-sections approximately 9 months post-injection from PBS- and AAV9-tMCK-CaMKII^CA^-injected paired muscles. (f) Muscle fiber minimum Feret diameter from 4 paired mice (1 male/3 female), approximately 9 months post-injection. Each point represents the median of approximately 200-300 fibers from one muscle. n = 4 muscles per treatment. Data were analyzed by paired Student’s t-test; the P value is shown in the graph. (g) Distribution of individual muscle fiber minimum Feret diameters pooled across the 4 muscles per treatment shown in panel (f).

SUPPLEMENTARY FIGURE 3 Short-term CaMKII^CA^ expression does not alter mitochondrial complex abundance or mtDNA copy number in TA muscles 7 weeks post-injection. (a) Western blot detection of representative subunits of mitochondrial complexes I–V. (+) denotes AAV9-CaMKII^CA^-injected TA, and (-) denotes AAV9-EGFP-injected TA. (b) Quantification of individual subunit expression levels relative to total protein staining. n = 7 EGFP-expressing muscles and n = 5 CaMKII^CA^-expressing muscles. Samples were from six mice: five mice (3 male, 2 female) expressed EGFP and CaMKII^CA^ in contralateral TA muscles, and one female mouse expressed EGFP in both TA muscles; multiple t-tests, nd = not a discovery (not significant). (c) Quantification of mitochondrial DNA to nuclear DNA copy number ratio by qPCR. n = 9 paired mice (4 male/5 female). Data were log-transformed and analyzed by paired Student’s t-test; P value is shown in the graph.

SUPPLEMENTARY FIGURE 4 Long-term CaMKII^CA^ expression does not change the abundance of mitochondrial complexes I-V in TA muscles approximately 9 months post-injection. (a) Western blot detection of representative subunits of mitochondrial complexes I-V. (+) denotes AAV9-tMCK-CaMKII^CA^-injected TA, and (-) denotes PBS-injected contralateral TA. (b) Quantification of individual subunit expression levels. n = 5 paired mice (2 male/3 female). Data were analyzed by paired multiple t-tests; nd, not a discovery (not significant).

SUPPLEMENTARY FIGURE 5 CollecTRI regulatory network analysis. (a) Activated and repressed transcription factors by CaMKII^CA^ expression (young CaMKII^CA^ vs. young EGFP). (b) Activated and repressed transcription factors by CN19o expression (old CN19o vs. old EGFP).
